# Chronic kidney disease among people living with HIV on TDF based regimen: A systematic review and meta-analysis

**DOI:** 10.1371/journal.pone.0318068

**Published:** 2025-02-06

**Authors:** Taklo Simeneh Yazie, Wondimeneh Shibabaw Shiferaw, Asaye Alamneh Gebeyehu, Assefa Agegnehu Teshome, Zenaw Debasu Addisu, Abebe Muche Belete

**Affiliations:** 1 Department of Pharmacy, Pharmacology and Toxicology, College of Health Sciences, Debre Tabor University, Debre Tabor, Ethiopia; 2 UQ Centre for Clinical Research, Faculty of Medicine, The University of Queensland, Herston, Australia; 3 Department of Nursing, Asrat Weldeyes Health Science Campus, Debre Berhan University, Debre Berhan, Ethiopia; 4 Department of Social and Public Health, College of Health Science, Debre Tabor University, Debre Tabor, Ethiopia; 5 Department of Biomedical Science, College of Health Sciences, Debre Tabor University, Debre Tabor, Ethiopia; 6 Department of Clinical Pharmacy, College of Health Sciences, Bahir Dar University, Bahir Dar, Ethiopia; 7 Department of Biochemistry, West African Centre for Cell Biology of Infectious Pathogens, Cell and Molecular Biology, University of Ghana, Accra, Ghana; 8 Department of Biomedical Science, Asrat Weldeyes Health Science Campus, Debre Berhan University, Debre Berhan, Ethiopia; Cairo University Kasr Alainy Faculty of Medicine, EGYPT

## Abstract

**Introduction:**

Chronic kidney disease is a major public health concern among people living with human immunodeficiency virus (PLWHIV) who are taking tenofovir disoproxil fumarate-based regimen. Despite the available evidence showing a high prevalence of CKD in this population, comprehensive pooled estimate of CKD among PLWHIV receiving TDF based regimen across the globe is lacking. Hence, the present systematic review aimed to provide a global pooled prevalence estimate of CKD.

**Method:**

We conducted a systematic review of literatures published between January 2000 and May 2024. Articles and grey literature were searched from the following databases and search engine: PubMed, EMBASE, Scopus, Web of science, The Cumulative Index to Nursing and Allied Health Literature (CINHAL), and Google Scholar. We included eligible studies that report magnitude of CKD in TDF based regimen. We executed the pooled CKD, subgroup analysis, and funnel plot using random effect model. All statistical analysis including sensitivity analysis were made using Stata 17 software.

**Results:**

Sixty-nine studies with 88299 participants included in this meta-analysis. The pooled prevalence of CKD was 7% (95% CI:6–8). CD4 count less than 200 copies per milliliter, and being female were associated with CKD.

**Conclusion:**

We concluded that the magnitude of CKD across the globe is high in people living with HIV who have received TDF based regimen. Early identification of CKD by considering regular renal function monitoring, and risk factors especially low CD4 count, and female gender at birth are essential.

**Trial registration:**

The protocol has been prospectively registered with PROSPERO ((CRD42020136813).

## Introduction

Chronic kidney disease (CKD) is a common complication among people living with HIV (PLWHIV) [1, 2]. Several evidence indicate that CKD is associated with the use of Tenofovir Disoproxil Fumarate (TDF) [3–7]. The distribution of CKD among PLWHIV on TDF varies across the globe: it ranges from 1–61.7% [8, 9]. A systematic review conducted by Cooper RD et al. in 2010 [10], and Mtisi TJ et al. in 2019 [11] confirmed the association of TDF utilization with the presence of kidney function loss. However, since then, there has been no recent comprehensive evidence elucidating the burden of CKD among PLWHIV on TDF.

Therefore, this review aims to determine the prevalence of CKD among PLWHIV receiving a TDF based regimen. Thus, the finding of this review will provide valuable insights into the management of people living with HIV patients on TDF and help policy makers and clinician to make informed decisions about the use of this drug in this patient’s population.

## Methods

This review was conducted following the guidance and instructions outlined in the Preferred Reporting Items for Systematic Review and Meta-analysis (PRISMA) [12] (S1 Table). The study protocol (CRD42020136813) was prospectively registered with PROSPERO. The chapter on systematic reviews of prevalence and incidence studies in Joanna Briggs Institute (JBI) Reviewer’s Manual for prevalence studies, and condition, context, and population (CoCoPop) was used to form the review questions [13]. Hence, this review’s research questions are: i) what is the prevalence of CKD among PLWHIV on TDF based regimen? ii) what factors contribute to CKD among PLWHIV on TDF?

### Eligibility criteria

Following CoCoPop framework, the eligibility criteria for the review are described as follows:

**Population:** We included studies involving participants (age 13 years or older) living with HIV and receiving TDF based regimen. These age group patients have comparable renal function [14] and TDF dose use [15]. We excluded studies involving participants with age less than 13 years.
**Condition:** We considered studies that report the main outcome of the study (i.e., prevalence of CKD among PLWHIV on TDF).
**Context:** We included studies conducted in community and institution-based studies that report the prevalence of CKD.
**Types of studies:** We included Clinical trial and observational studies (cross sectional, cohort (retrospective, and prospective). We excluded case series, and case reports. We included studies published from January 1, 2000 to May 2, 2024. We considered only studies published in English.

### Information source

To conduct this review, the search strategy employed was the Peer Review of Electronic Search Strategies (PRESS) methodology for systematic reviews in our search strategy [16]. To undertake the search strategy, the primary investigators initially developed search terms. Subsequently, all co-authors reviewed and approved the comprehensive search terms. The databases used were PubMed, Scopus, EMBASE, Cumulative Index to Nursing and Allied Health Literature (CINHAL), and Web of Science. Additionally, we examined reference lists in papers for relevant papers. Furthermore, we searched papers from grey literature like Google scholar.

#### Search strategies

Initial key words used were HIV, Chronic kidney disease, and TDF. Following this, independent search terms were developed for each key words, for HIV included “HIV” OR “hiv” OR “human immunodeficiency virus” OR”AIDS “OR”acquired immunodeficiency syndrome.” Search terms for chronic kidney disease include **“**Chronic Kidney Failure” OR “Chronic Renal Failure” OR “Chronic kidney disease” OR “End-Stage Kidney Disease” OR “End-Stage Renal Disease” OR “End-Stage Renal Failure” OR “ESRD” OR “Renal Insufficiency” OR “Renal impairment” OR “Kidney impairment” OR “Renal failure” OR “Kidney failure” OR “Renal dysfunction” OR “Kidney dysfunction.” Search terms for TDF includes “Tenofovir” OR “Tenofovir Disoproxil Fumarate” OR “TDF.” The full search term is in included in the (S2 Table).

#### Selection of studies

Initially, the articles found from each database were imported into Endnote version 8.1 citation manager software. Duplicate articles were then removed. Following this, the titles and abstracts of each article were assessed for inclusion by Two (TSY and AMB) independent review authors. Additionally, articles deemed suitable for the full-text review were evaluated for inclusion against the pre-identified inclusion criteria by other two review co-authors (AAG and WSS). Any disagreements arising during the selection process were resolved with consultation of a third review author (ZDA).

#### Methodological quality assessment

The included studies were evaluated methodological quality using the Newcastle-Ottawa Scale (NOS) tool for observational [17], and version 2 of the Cochrane risk-of-bias tool for randomized trials (RoB 2) for randomized controlled trial studies [18]. In each included studies we assessed representativeness, response rate, method, comparability of the subject and the appropriateness of statistically analysis used for observational studies, and randomization, allocation concealment, adherence to intervention, and outcome assessment for clinical trials. Two review authors (WSS and AAT) checked quality of the studies using the above criteria. Any disagreement was resolved through discussion (S3 Table).

#### Data extraction

We used the Joanna Briggs Institute (JBI) extraction form for prevalence and incidence studies available in Munn et al [13]. Two authors (AAG and WSS) performed data extraction. The following study characteristics were extracted from included studies: first author, publication year, region, study design, sample size, and outcome reported (i.e. CKD) and associated factors like age, sex, CD4 count, and glomerular filtration rate. We resolved disagreements by consensus or discussion.

### Missing data handling

In this review, we used a complete-case analysis approach to include only studies that had complete data for the outcome of interest. For studies with missing data, we tried to contact the corresponding authors to obtain the missing information. However, despite these efforts, none of them responded to our requests.

#### Approaches to CKD diagnosis

There is no internationally standardized eGFR estimation method recommended to be used universally across the world. Hence, studies used different eGFR estimation method to assess renal function. These are Cockcroft-Gault (CG) [19], Modification of Diet in Renal Diseae (MDRD) [20], Chronic Kidney Disease Epidemiology (CKD-EPI) [21], Modification of Diet in Renal Disease (MDRD) without race factor [22], and Japanese Society (JSE) [23]. The presence of proteinuria or kidney damage confirmed via imaging alone or eGFR<60ml/min persisted for at least 3 months can be used to diagnose CKD [24]. Treatment guidelines recommend eGFR or CrCl <50 [15] or 60ml/min [25, 26] to define CKD, and to modify or avoid TDF use in HIV care, so we included studies that used eGFR or CrCl level of either <50 or 60 ml/min. CrCl/eGFR<50/60ml/min occurred onspot or persisted at least 3 months post TDF initiation was used as CKD diagnostic criterion to loosen the inclusion criteria for the purpose of revealing the pooled estimate of CKD across the globe.

#### Assessment of risk of bias

Each included study was evaluated using Hoy risk of bias assessment tool for reporting prevalence data [27]. The Hoy score is marked out of ten and a value of 8–10 indicated low bias, 5–7 moderate bias and ≤4 high bias. Two review authors (AAG and TSY) independently assessed the risk of bias.

#### Heterogeneity and publication bias

We used Cochran’s *Q* and *I* ^2^ statistics to measure heterogeneity among the studies included in each analysis [28]. Higgins et al. suggest that an I^2^ value of 25%, 50%, and 75% indicate low, medium, and high heterogeneity, with respective order. We performed subgroup analysis based on region (continent), CKD diagnostic criteria, income level, and study design. We also performed sensitivity analysis was also conducted for each study’s effect on the overall prevalence. Funnel plot was used to visually inspect publication bias. Egger’s test was used to assess statistical significance of publication bias [29].

#### Statistical analysis

DerSimonian–Laird random-effects models [30] was used to generate the pooled prevalence of CKD. The pooled effect size (i.e., prevalence) with weighted and their 95% confidence interval (CI) was generated. Additionally, the pooled effect size (i.e., odds ratio for age, and eGFR; hazard ratio for sex, and CD4 count) also generated with their 95% CI. We displayed all analysis in the form of forest plot. We used Stata software version 17.

## Results

### Description of included studies

#### Search results

**Fig 1:**
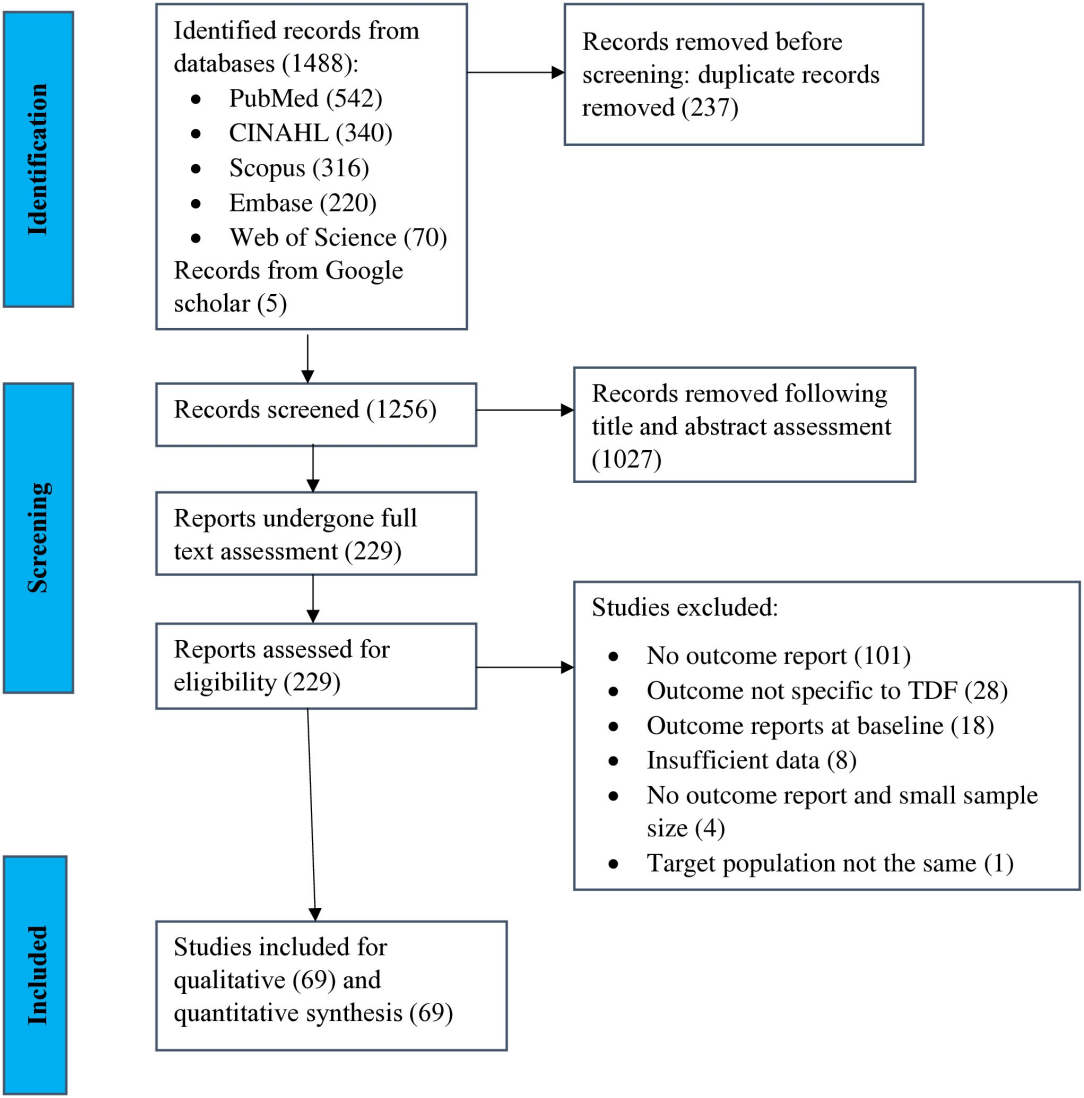
PRISMA flow diagram describing the selection processes of eligible studies.

We access 1493 studies from databases and manual search. After removal of duplicates, 1256 studies remained, and we excluded 1027 studies during title and abstract screening stage. We reviewed the remaining 229 studies for full-text eligibility, and excluded 160 due to various reasons (Fig 1).

### Characteristics of included studies

We included sixty-nine studies comprising of 88299 study participants in the analysis. Among them, three clinical trial [31–33], fifteen prospective cohort [20–22, 34–45], thirty-five retrospective cohort [6, 8, 9, 19, 23, 46–75], and sixteen cross sectional [76–91]. The sample size in the included studies ranges from 38 [61] to 11153 [45]. According to the World Bank geographical classification [92], we found twenty-four studies in East Asia & Pacific, twenty-one in Sub-Saharan Africa, thirteen in Europe & Central Asia, eight in North America, and three in more than one region. We reviewed studies conducted: seven each from Japan, and US; four each from Ethiopia, China, Ghana, Italy, Spain, and Thailand; three each from Australia, and France; two each from South Africa, Cameroon, Nigeria, South Korea, United Kingdom, and Namibia; and one study each in Malawi, Eastern and southern African countries, Uganda, Malaysia, India-UK, Myanmar, Zambia, Singapore, Canada and New Zealand, Tanzania, Asian countries, and Vietnam. The included study characteristics including study region, sample size, and eGFR estimation methods are presented in Table 1 below.

**Table 1:** Characteristics of included studies.

Author-year	Continent	Study design	Sample size	eGFR equation	eGFR cutoff point
Cournil A et al. 2017 [32]	Sub-Saharan Africa	RCT	275	MDRD	<60ml/min
Chikwapulo B et al., 2018 [19]	Sub-Saharan Africa	RetroCT	426	CG	<50ml/min
Yazie TS et al., 2019 [35]	Sub-Saharan Africa	ProCohort	63	CKD-EPI	<60 ml/min
Mwafongo A et al., 2015 [31]	Sub-Saharan Africa	RCT	741	CG	<50 ml/min
Zachor H et al., 2016 [50]	Sub-Saharan Africa	RetroCT	650	CKD-EPI	<60 ml/min
Ojen BV et al., 2018 [49]	Sub-Saharan Africa	RetroCT	3214	MDRD	<60 ml/min
Nartey ET et al., 2019 [51]	Sub-Saharan Africa	RetroCT	300	CG	<50 ml/min
Nyende L et al., 2020 [79]	Sub-Saharan Africa	CS	278	CKD-EPI	< 60ml/min
Neary M et al., 2020 [34]	Sub-Saharan Africa	ProCohort	66	CG	<60mL/min
Bock P et al., 2019 [47]	Sub-Saharan Africa	RetroCT	1634	MDRD worf	< 60 mL/min
Belete AM et al., 2021 [77]	Sub-Saharan Africa	CS	243	CKD-EPI	<60 ml/min
Debeb SG et al., 2021 [48]	Sub-Saharan Africa	RetroCT	200	CKD-EPI	<60 ml/min
Fritzsche C et al.,2017 [76]	Sub-Saharan Africa	CS	119	CKD EPI	< 60/mL/min
Chadwick D et al., 2015 [78]	Sub-Saharan Africa	CS	101	CG	<60 ml/min
Kim JH et al., 2022 [39]	East Asia & Pacific	ProCohort	392	MDRD	< 60/mL/min
Nishijima T et al., 2016 [38]	East Asia & Pacific	ProCohort	417	CKD-EPI	<60 ml/min
Young et al., 2007 [40]	North America	ProCohort	593	CG	<50 ml/min
Feng L et al., 2022 [20]	East Asia & Pacific	ProCohort	622	MDRD	<60 ml/min
Sutton SS et al., 2020 [60]	North America	RetroCT	4475	CKD-EPI	<60 ml/min
Cheung J et al., 2018 [21]	East Asia & Pacific	ProCohort	985	CKD-EPI	<60 ml/min
Tan LKK et al., 2009 [61]	Europe & Central Asia	RetroCT	38	MDRD	<60 ml/min
Milazzo L et al., 2016 [62]	Europe & Central Asia	RetroCT	78	CKD-EPI	<60 ml/min
Kalemeera F et al., 2020 [52]	Sub-Saharan Africa	RetroCT	6744	CKD-EPI	<50 ml/min
Calza L et al., 2014 [84]	Europe & Central Asia	CS	409	MDRD	<60 ml/min
Quesada PR et al., 2015 [41]	Europe & Central Asia	ProCohort	451	MDRD	<60 ml/min
Low JZ et al., 2018 [63]	East Asia & Pacific	RetroCT	314	MDRD	<60 ml/min
Pujari SN., 2014 [53]	South Asia-Europe & Central Asia	RetroCT	1225	MDRD	<60 ml/min
Jotwani V et al., 2016 [81]	North America	CS	573	CKD-EPI	<60 ml/min
Visuthrankul J et al., 2021 [54]	East Asia & Pacific	RetroCT	700	MDRD	<60 ml/min
Nishijima T et al., 2014 [36]	East Asia & Pacific	ProCohort	422	JSN equation	<60 ml/min
Kyaw NTT et al., 2015 [56]	East Asia & Pacific	RetroCT	1372	CG	<50 ml/min
O’Donnel EP et al., 2011 [55]	North America	RetroCT	348	MDRD	<60 ml/min
Nishijima T et al., 2011 [57]	East Asia & Pacific	RetroCT	495	MDRD	<60 ml/min
Okpa HO et al., 2019 [80]	Sub-Saharan Africa	CS	60	CG	<60 ml/min
Woolnough EL et al., 2018 [58]	East Asia & Pacific	RetroCT	473	CKD-EPI	<60 ml/min
Nishijima T et al., 2017 [82]	East Asia & Pacific	CS	774	JSN equation	<60 ml/min
Obiri-Yeboah D et al., 2018 [83]	Sub-Saharan Africa	CS	288	MDRD	<60 ml/min
Lapadula G et al., 2016 [37]	Europe & Central Asia	ProCohort	2023	CKD-EPI	<60 ml/min
Hsu R et al., 2020 [59]	North America	RetroCT	6222	CKD-EPI	<60 ml/min
Morlat P et al., 2013 [22]	Europe & Central Asia	ProCohort	3268	MDRD worf	<60 ml/min
Chabala FW et al., 2021 [42]	Sub-Saharan Africa	ProCohort	201	CKD-EPI	<60 ml/min
Likanonsakul S et al.,2016 [85]	East Asia & Pacific	CS	273	CKD-EPI	<60 ml/min
Flandre P et al.,2016 [43]	Europe & Central Asia	ProCohort	3543	MDRD worf	<60 ml/min
Lee KH et al.,2017 [65]	East Asia & Pacific	RetroCT	50	MDRD worf	<60 ml/min
Paengsai N et al.,2022 [66]	East Asia & Pacific	RetroCT	8710	CKD-EPI	<60 ml/min
Suzuki S et al.,2017 [6]	East Asia & Pacific	RetroCT	720	CKD-EPI	<60 ml/min
Campbell LJ et al.,2009 [46]	Europe & Central Asia	RetroCT	843	MDRD	<60 ml/min
Domingo P et al.,2019 [64]	Europe & Central Asia	RetroCT	4852	CKD-EPI	<60 ml/min
Ando M et al., 2011 [44]	East Asia & Pacific	ProCohort	244	JSN equation	<60 ml/min
Chua AC et al., 2012 [67]	East Asia & Pacific	RetroCT	154	CG	<50 ml/min
Nishijima T et al., 2015 [23]	East Asia & Pacific	RetroCT	703	JSN equation	<60 ml/min
Ahmed E et al., 2020 [86]	Sub-Saharan Africa	CS	290	CG	<60 ml/min
Chan A et al., 2019 [33]	North America-East Asia & Pacific	OLCT	335	CG	<50 ml/min
Huang Y et al., 2017 [8]	East Asia & Pacific	RetroCT	391	CKD-EPI	<60 ml/min
Juega-Mariño J et al., 2017 [87]	Europe & Central Asia	CS	699	MDRD	<60 ml/min
Mwemezi O et al., 2020 [88]	Sub-Saharan Africa	CS	249	CKD-EPI	<60 ml/min
Reynes J et al., 2013 [89]	Europe & Central Asia	CS	658	MDRD	<60 ml/min
Monteagudo-Chu et al., 2012 [69]	North America	RetroCT	111	MDRD	<60 ml/min
Medland NA et al., 2017 [71]	East Asia & Pacific	RetroCT	1442	CKD-EPI	<50 ml/min
Suppadungsuk S et al., 2022 [9]	East Asia & Pacific	RetroCT	141	CKD-EPI	<60 ml/min
Calza L et al.,2013 [68]	Europe & Central Asia	RetroCT	235	MDRD	<60 ml/min
Pedrol E et al., 2015 [72]	Europe & Central Asia	RetroCT	73	CKD-EPI	<60 ml/min
Kalemeera F et al., 2023 [73]	Sub-Saharan Africa	RetroCT	7526	CG	<60 ml/min
Joshi et al., 2019 [74]	South Asia-East Asia & Pacific	RetroCT	703	CKD-EPI	<60 ml/min
Hoang C et al., 2020 [90]	East Asia & Pacific	CS	400	CKD-EPI	<60 ml/min
Mocroft A et al., 2015 [45]	Middle East &North America, Latin America & Caribbean, North America, East Asia & Pacific, Europe & Central Asia	ProCohort	11153	CG	<60 ml/min
Crum-Cianflone N et al., 2010 [91]	North America	CS	318	MDRD	<60 ml/min
Liu F et al., 2021 [75]	East Asia & Pacific	RetroCT	797	MDRD worf	<60 ml/min
Yang J et al., 2019 [70]	East Asia & Pacific	RetroCT	414	MDRD	<60 ml/min

**Abbreviations:** CS: Cross sectional; ProCohort: Prospective cohort; RetroCT: Retrospective cohort; RCT: Randomized controlled trial; OLCT: Open label clinical trial; MDRD: Modification of diet in renal disease equation; CKD-EPI: Chronic kidney disease epidemiology collaboration equation; MDRD worf: MDRD equation without race factor; CG: Cockcroft-Gault; JSN equation: Japan society of nephrology equation

### Prevalence of chronic kidney disease

**Fig 2:**
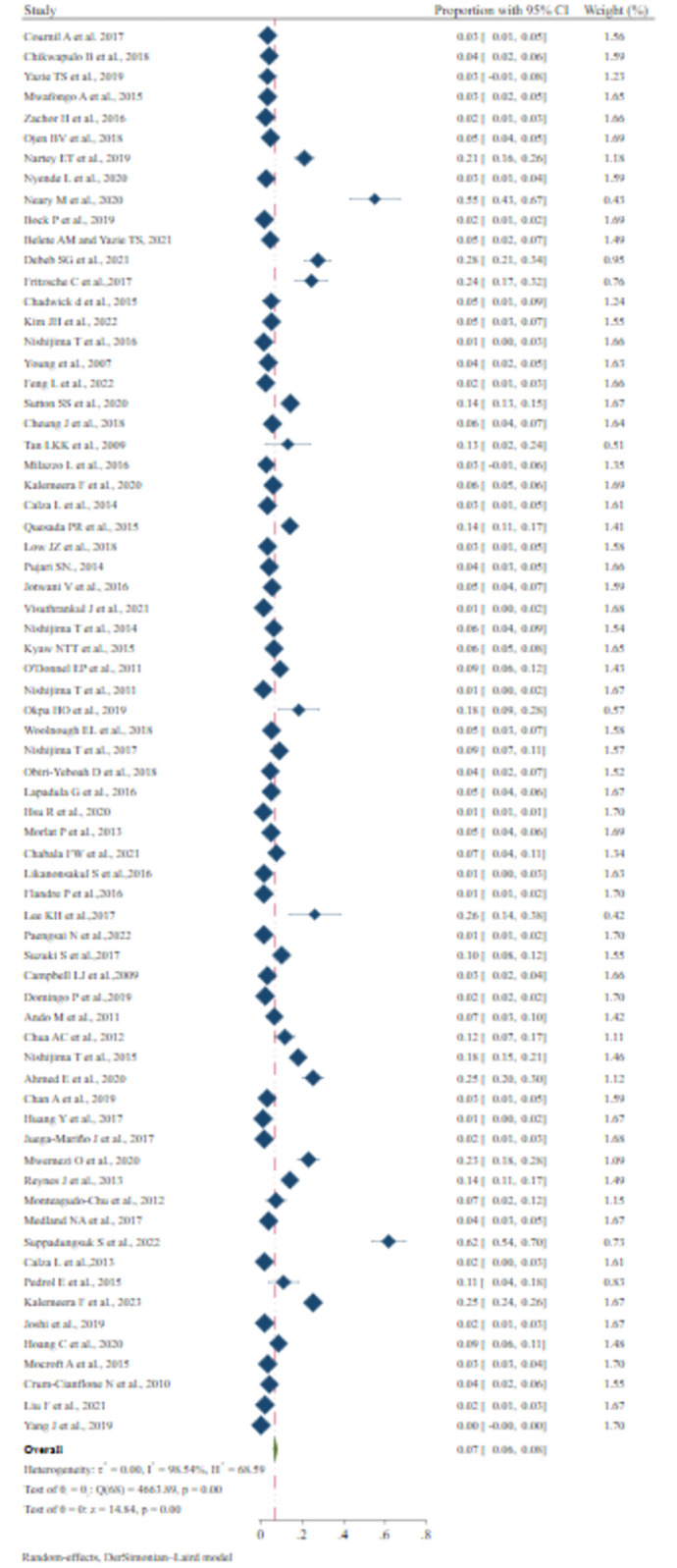
Overall pooled proportion of included studies.

In the present meta-analysis, we used sixty-nine studies to estimate the pooled prevalence of CKD. Based on Hoy D et al., we found three (4.4%) studies with moderate risk of bias, and sixty-six (95.5%) studies with low risk of bias. The overall pooled prevalence of CKD diagnosed with estimated glomerular filtration rate (eGFR) was 7% (95% CI: 6%-8%), I^2^ = 98.54%, p < 0.01 (Fig 2). This I squared result showed high heterogeneity among studies, which indicates the necessity of subgroup analysis.

### The prevalence of CKD by its diagnostic crteria

All but two included studies used only estimated glomerular filtration rate (eGFR) or creatine clearance (CrCl)<50/60ml/min to diagnose CKD. Two studies used proteinuria in addition to eGFR or CrCl<50/60ml/min to estimate CKD [44, 88].

In this meta-analysis, according to the eGFR cutoff point of <50 versus <60ml/min/average adult body surface area, the pooled estimate of CKD was 6% (95% CI: 4–7%), and 7% (95% CI: 6–8%), respectively. Heterogeneity between studies in both groups were found to be high; however, regarding the pooled CKD etstimate, there was no significant difference (P = 0.19) between groups.

In the present review concerning eGFR or CrCl <50/60ml/min persistence to define CKD, thirty five studies used on the spot estimation (eight with no baseline eGFR data and twenty seven with normal baseline eGFR), while thirty four studies used a two time point estimation at least 3 months apart. According to these diagnostic criteria, the pooled prevalence of CKD was 9% (95% CI: 6–12%) diagnosed with on the spot eGFR with no baseline data, 9% (95% CI: 7–11%) confirmed with on the spot eGFR with normal baseline data, and 5% (95% CI: 4–6%) in cases with <50 or 60ml/min confirmed at least 3 months apart. We found high heterogeneity and significant difference between studies in all subgroups and between groups.

Regarding eGFR estimation equations, among the included studies, 27 used CKD-EPI, 20 used MDRD, 13 used CG, 5 used MDRD without race factor, and 4 used JSE equation to estimate eGFR. In subgroup analysis of the pooled prevalence of CKD based on eGFR estimation equations, we found a prevalence of 13% (95% CI: 8–18%) in studies that used CG, 10% (95% CI: 5–15%) in studies that used JSE, 7% (95% CI: 5–8%) in those using CKD-EPI, 4% (95% CI: 3–6%) with MDRD, and 3% (95% CI: 1–5%) with MDRD without race factor. In these subgroup analyses, we found high heterogeneity between studies in all groups, with significant group differences. Additionally, we found that pooled prevalence of CKD based on CG, MDRD, and MDRD without race factor was significantly different from the overall pooled prevalence estimate of CKD (Table 2).

**Table 2:** CKD based on CKD diagnostic approach.

Variable	Category of variable	Pooled estimate of CKD (95% CI)	tau2	% I2	H2	df	Q	P	Test of group differences
**eGFR cutoff point**	<50ml/min	0.057(0.042–0.073)	0.000	91.13	11.28	8	90.20	0.000	Chi2(1) = 1.74, P = 0.187
	<60ml/min	0.070(0.060–0.080)	0.001	98.65	74.06	59	4369.81	0.000	
**eGFR<50/60ml/min confirmation**	On the spot^a^	0.088(0.055–0.120)	0.002	93.01	14.32	7	100.21	0.000	Chi2(2) = 12.23, P = = 0.002
	On the spot^b^	0.088(0.067–0.109)	0.003	99.21	126.40	26	3286.40	0.000	
	=/>3month	0.053(0.043–0.062)	0.001	97.36	37.84	33	1248.72	0.000	
**eGFR estimation**	CG	0.131(0.079–0.183)	0.009	99.38	160.80	12	1929.62	0.000	Chi2(4) = 26.71, P = 0.000
	MDRD	0.044(0.03–0.056)	0.001	95.86	24.13	19	458.51	0.000	
	CKD-EPI	0.066(0.054–0.079)	0.001	98.05	51.21	26	1331.46	0.000	
	MDRD worf	0.029(0.012–0.045)	0.000	95.26	21.08	4	84.32	0.000	
	JSE	0.099(0.050–0.148)	0.002	93.52	15.42	3	46.26	0.000	

**Abbreviation**: MDRD: Modification of diet in renal disease equation; CKD-EPI: Chronic kidney disease epidemiology collaboration equation; MDRD worf: MDRD equation without race factor; CG: Cockcroft-Gault; JSN equation: Japan society of nephrology equation.

^a^On the spot CKD confirmation without baseline eGFR,

^b^On the spot CKD confirmation with normal baseline eGFR.

#### CKD in PLWHIV by study design, age group, region and income

The pooled prevalence of CKD based on study design was as follows: 9% (95% CI: 6–11%) in cross sectional studies, 7% (95% CI: 6–9%) in retrospective cohort studies, 5% (95% CI: 4–6%) in prospective cohort studies, and 3% (95% CI: 2–4%) in clinical trials. We found high heterogeneity accross all study groups except clinical trials. Pooled prevalence of CKD in prospective cohort studies, and clinical trials were significantly different from the overall pooled prevalence. We found significant difference between the study design groups.

Among the included studies, six were conducted in low-income countries (LIC), thirteen in lower middle-income countries (LMIC), twelve in upper middle-income countries (UMIC), thirty-five in high income countries (HIC), and three in others (from LIC (n = 1), LMIC, UMIC, and HIC (n = 2)). These studies showed high heterogeneity, with significant difference between income groups. Studies from LMIC, and those spanning multiple income categories showed a significant difference in pooled CKD prevalence compared to the overall pooled prevalence of CKD.

We included twelve studies with participants aged thirteen years and above, and fifty-seven studies with participants aged eighteen years and older. Subgroup analysis did not show significant difference in pooled prevalence of CKD between these age groups. We found 9% (95% CI: 6–13%), and 6% (95% CI: 5–7%) pooled prevalence of CKD in age groups of thirteen years and above, and eighteen years and older group, respectively.

The highest pooled prevalence of CKD was found in Sub-Sahara Africa (11.7% [95% CI: 8.4–15%]), while the lowest was in the others regions group (3% [95% CI: 2–4.1%]). We found significantly different pooled prevalence of CKD in Sub-Sahara Africa, and others regions group compared to the overall pooled prevalence of CKD. We found high heterogeneity between studies (P<0.05)) from all group of regions. We also found significant difference between region groups (Table 3).

**Table 3:** CKD in PLWHIV by study design, age group, region and income.

Variable	Category of variable	Pooled estimate of CKD (95% CI)	tau^2^	% I^2^	H^2^	df	Q	P	Test of group differences
**Study design**	Cross sectional	0.086 (0.061–0.110)	0.002	94.73	18.97	15	284.57	0.000	Chi2(3) = 33.13, P = 0.000
	Retro cohort	0.073(0.059–0.087)	0.002	99.15	117.04	34	3979.38	0.000	
	Prospective cohort	0.051(0.039–0.063)	0.000	94.86	19.46	14	272.48	0.000	
	Clinical trial	0.032(0.023–0.041)	0.000	0.00	1.00	2	0.06	0.971	
**Income level**	Low	0.106(0.047–0.164)	0.005	95.91	24.44	5	122.21	0.000	Chi2(4) = 47.44, P = 0.000
	Lower middle	0.109(0.083–0.135)	0.002	94.19	17.21	12	206.55	0.000	
	Upper middle	0.071(0.044–0.098)	0.002	99.62	260.99	11	2870.84	0.000	
	High	0.058(0.047–0.069)	0.001	97.08	34.21	34	1163.09	0.000	
	Other	0.028(0.018–0.038)	0.000	75.17	4.03	2	8.05	0.018	
**Age**	>/ = 13 years	0.092(0.058–0.125)	0.003	99.62	263.54	11	2898.89	0.000	Chi2(1) = 2.91, P = 0.088
	>/ = 18 years	0.061(0.053–0.069)	0.001	96.81	31.39	56	1757.89	0.000	
**Region**	Sub-Sahara Africa	0.117(0.084–0.150)	0.005	99.02	101.93	20	2038.58	0.000	Chi2(4) = 29.81, P = 0.000
	East Asia & Pacific	0.056(0.045–0.067)	0.001	97.24	36.17	23	832.01	0.000	
	Europe & Central Asia	0.047(0.034–0.060)	0.000	94.98	19.93	12	239.21	0.000	
	North America	0.059(0.016–0.103)	0.004	98.88	89.12	7	623.82	0.000	
	Other	0.030(0.020–0.041)	0.000	80.36	5.09	2	10.18	0.006	

#### Meta-analysis of factors associated with CKD

**Fig 3:**
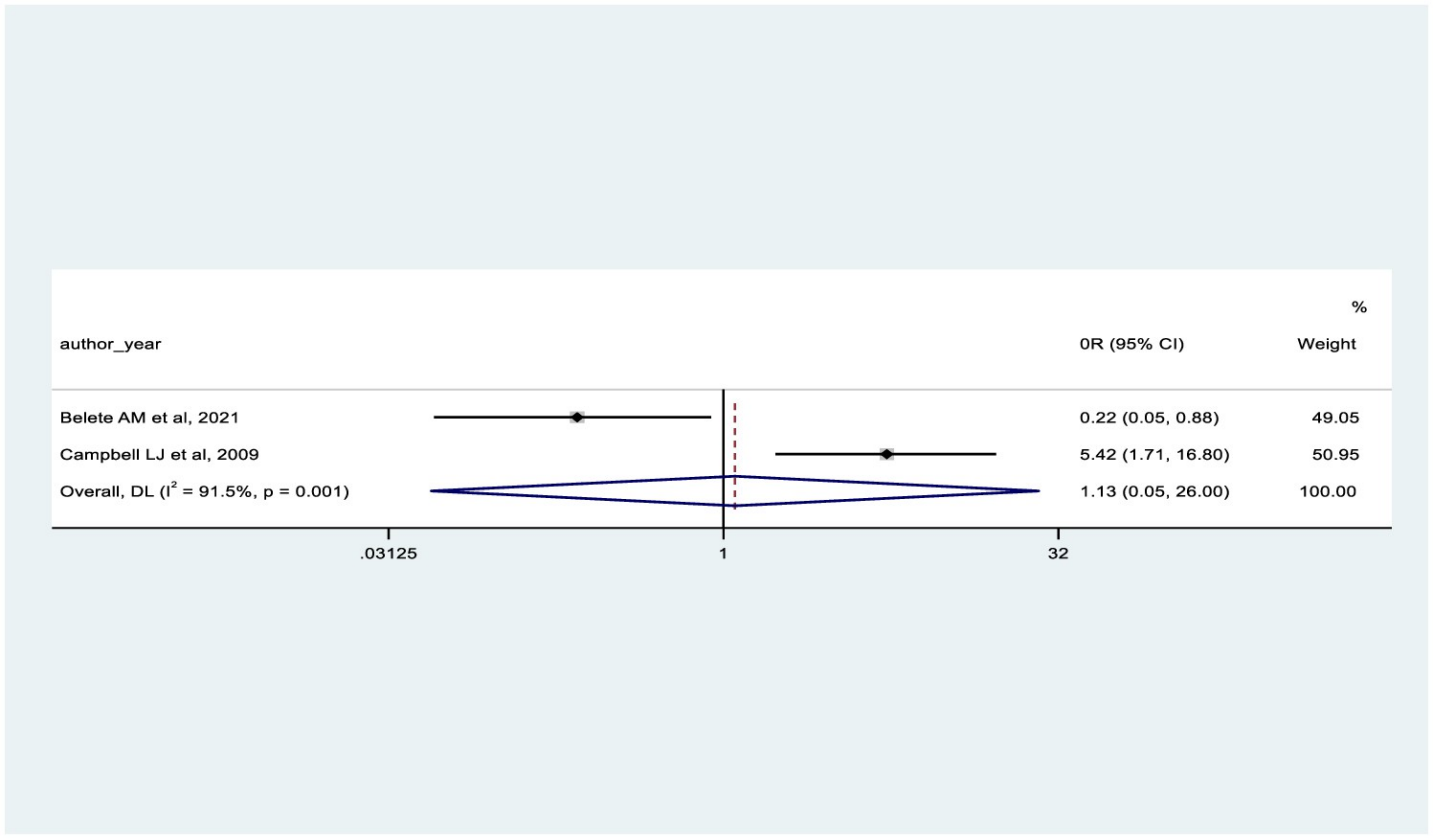
Shows the forest plot on the association between age of participants with presence of CKD.

**Fig 4:**
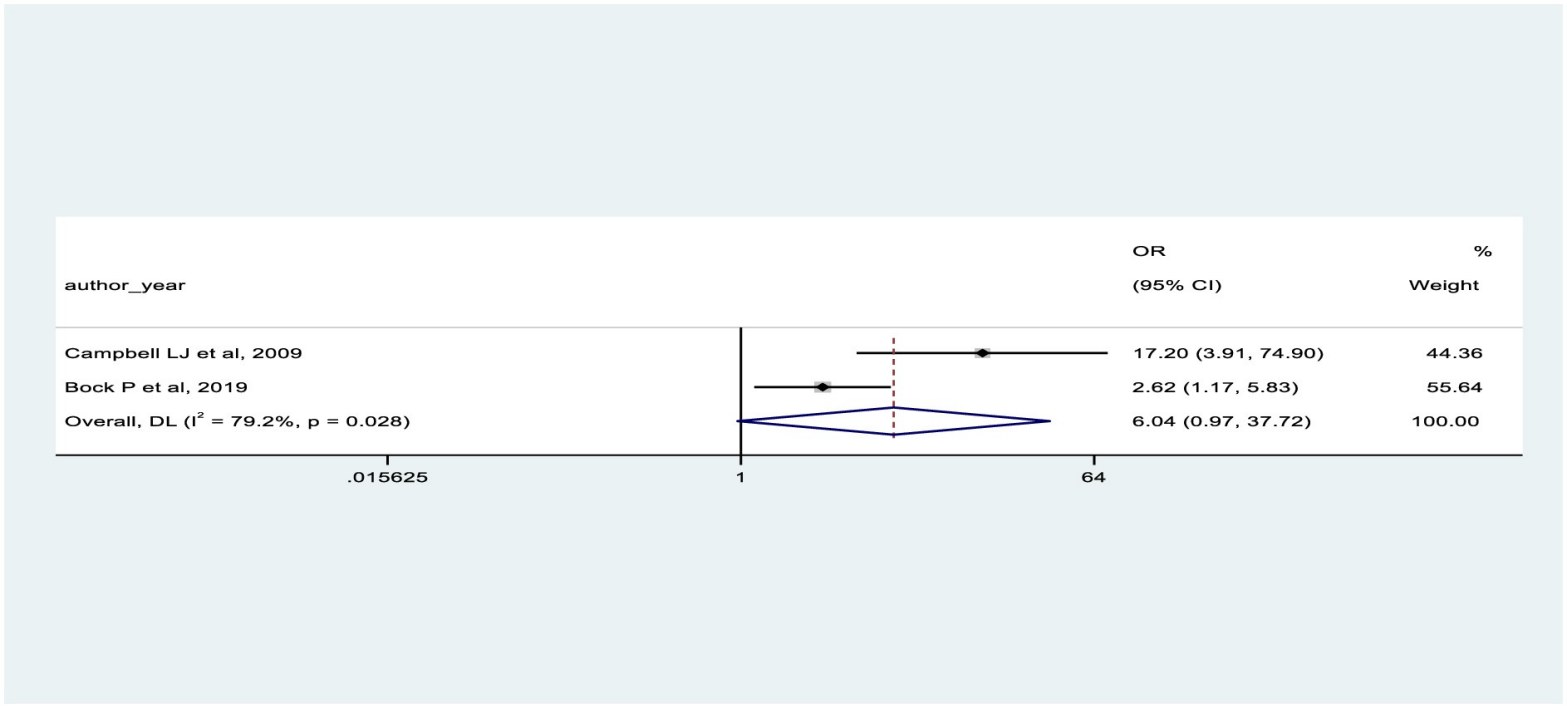
Shows the forest plot on association between eGFR 60–89 and presence of CKD.

In our meta-analysis, we used six studies to determine the pooled effect of predictor variables. We reported the pooled odds effect for age >50 years (OR = 1.13, 95% CI: 0.05–26.00) [46, 77], and eGFR in the range of 60-79ml/min (OR = 6.04, 95% CI: 0.97–37.72) [46, 47], each based on two studies. These two factors did not show a significant association with CKD (Figs 3 and 4).

**Fig 5:**
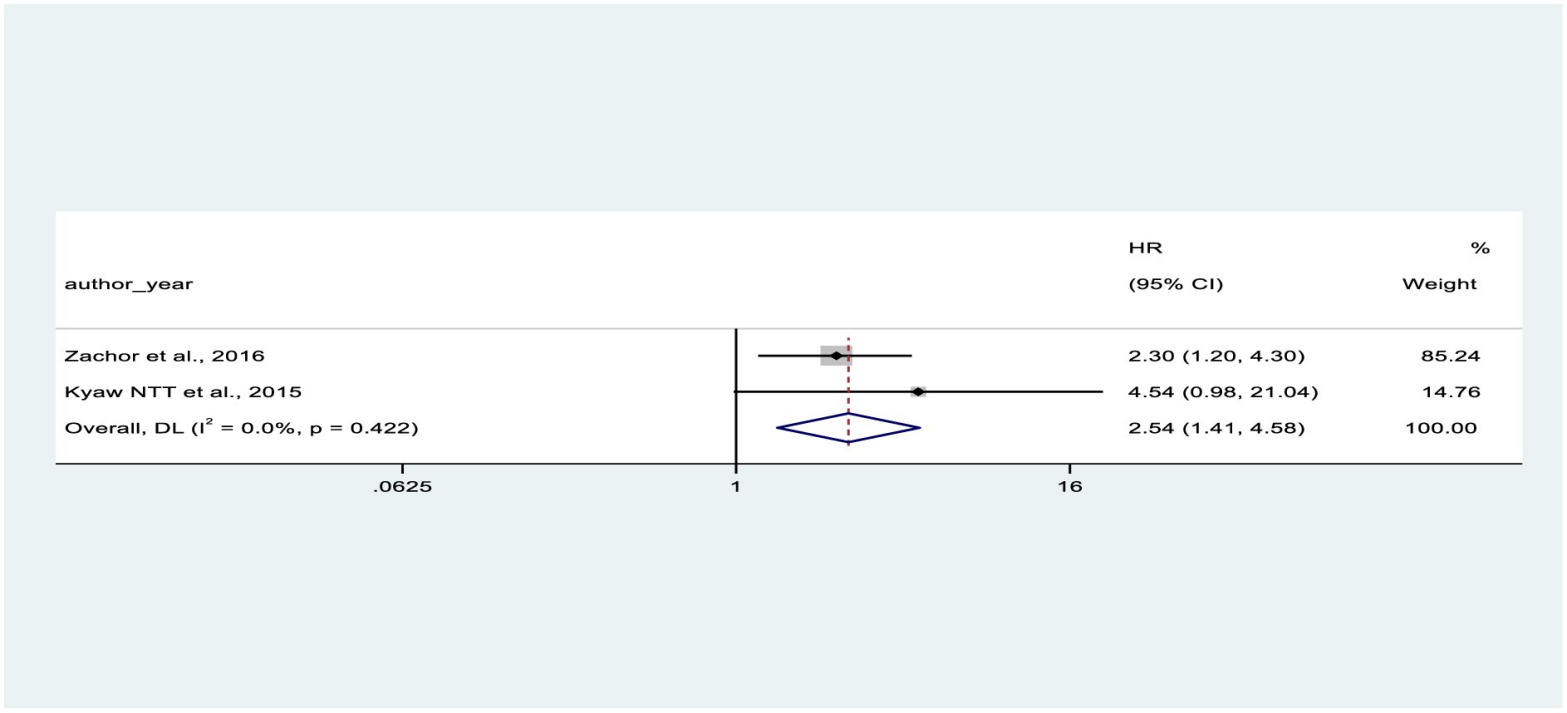
Shows the forest plot on association between CD4 count of study participants and CKD.

**Fig 6:**
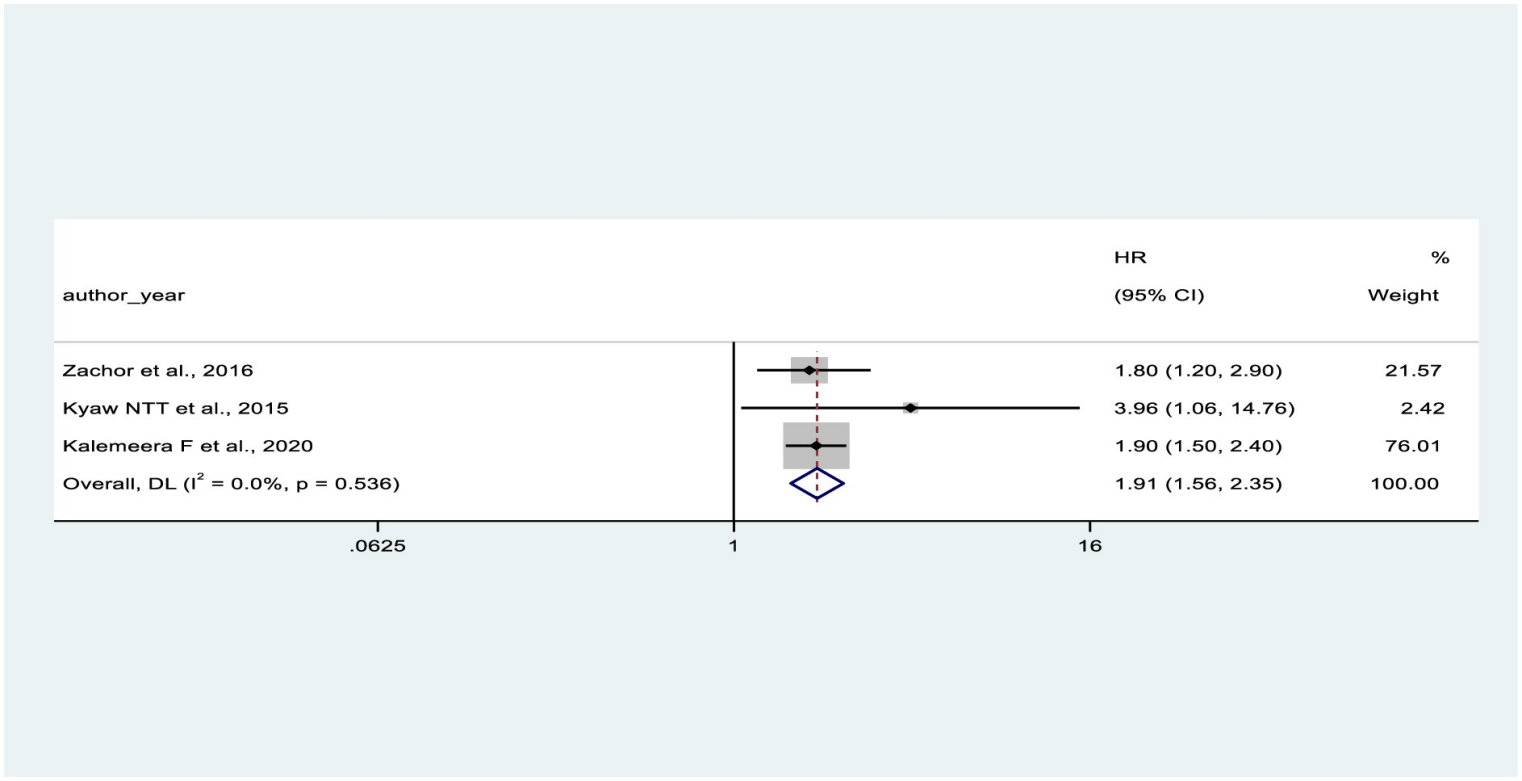
The forest plot on the association between gender at birth on CKD.

In the present meta-analysis, we found a pooled hazard ratio of CD4 count less than 200 (HR = 2.54, 95% CI: 1.41–4.58) compared with higher CD4 counts in two studies [50, 56], and of being female (HR = 1.91, 95% CI: 1.56–2.35) compared with being male in three studies [50, 52, 56]. These factors were significantly associated with CKD (Figs 5 and 6).

### Description of factors associated with CKD in included studies

Several factors were associated with CKD in the included studies; however, we did not present their pooled effect because the studies used different category of independent factors, different statistical analysis methods or both.

#### Age

Regarding age, different statistical methods were used to determine the association between age and the presence of CKD. Participants aged 42–53 years, compared to those aged 18–29 years (OR = 3.1, [95% CI: 1.12–8.55]) [86], and those older than 45 years, compared to 18–35 years (OR = 3.25. 95% CI: 1.3–8.14 [47], were three times more likely to experience CKD. Study participants had almost 4% increased risk of developing CKD (RR = 1.05, 95% CI: 1.01–1.09) [19];(RR = 1.04, 95% CI: 1.03–1.06) [51] for every one year increase in age. Participants older than 45 years were three times more likely to have CKD compared to those aged 18–35 years (HR = 3.4, 95% CI: 2.2–5.2) [56], while an increase in age by ten years was associated with almost twice the risk of experiencing CKD (HR = 2.21, 95% CI: 1.61–3.05(53); HR = 1.9, 95% CI: 1.1–3.29(50)).

#### Body mass index (BMI)

BMI was a significant predictor of CKD in some included studies. Study participants with a lower BMI were positively associated with the presence of CKD compared to their counterparts. Specifically, a BMI of <18.5 kg/m^2^ compared to >/ = 18.5 kg/m^2^ (OR = 4.39, 95% CI: 2.24–8.61) [86], <18.5 kg/m^2^ compared to 18.5–24.9 kg/m^2^ (RR = 3.87, 95% CI: 2.49–6.03) [51], a BMI of <16 kg/m^2^ (HR = 2.3, 95% CI: 1.1–5), and 16–18.5 (HR = 1.8, 95% CI: 1.1–3.2) compared to 18.5–24.9 kg/m^2^ [56] were associated with CKD.

#### World Health Organization (WHO) clinical stage

Concerning the WHO HIV/AIDS clinical stages, the risk of experiencing CKD was almost three times for individuals in stage III (RR = 3.78, 95% CI: 1.42–10.06), and stage IV (RR = 3.42, 95% CI: 1.16–10.9) compared to those in stage I [51]. On the other hand, another study found a negative association between clinical stage IV and the presence of CKD compared to stage I (OR = 0.1, 95% CI: 0.03–0.36 [86].

#### Comorbidity

The presence of high diastolic blood pressure, cancer, and diabetes mellitus as comorbidities were positive predictors of the presence of CKD compared to their counterparts. specifically, DBP >100 mmHg (RR = 2.78, 95% CI: 1.02–7.58) [19], cancer (OR = 18.2, 95% CI: 122–271.7) [77], and DM (HR = 3.6, 95% CI: 1.6–8.2) [56] were significantly associated with the presence of CKD.

#### Antiretroviral drug class, prior ART exposure, and viral load

Regarding the antiretroviral drug class, participants who have received TDF with ritonavir boosted protease inhibitors (PI/r) had higher risk of developing CKD compared to those on other regimens (HR = 2.4, 95% CI: 1.21–4.7) [53]. In contrast, NNRTIs were found to be 55% protective against CKD development compared to PI/r (OR = 0.45, 95% CI: 0.24–0.83) [41]. Prior antiretroviral therapy exposure (OR = 1.22, 95% CI: 1–1.5) [46], and longer duration of TDF exposure (OR = 26.3, 95% CI: 2.02–343.04 [86]; OR = 1.16, 95% CI: 1.04–1.3) [41] were positive predictor of CKD development. Additionally, a viral load of HIV RNA per 1log10 copies/ml higher was associated with almost four times the risk of developing CKD compared to their counterparts (OR = 4.41, 95% CI: 1.65–11.78) [31].

#### Baseline renal function

Baseline eGFR 60-89ml/min (HR = 1.7, 95% CI: 1.2–23) compared to eGFR >/ = 90ml/min [52] showed a significant association with CKD. Moreover, studies revealed significant associations between creatinine clearance per 10ml increase (OR = 0.8, 95% CI: 0.64–0.99), creatinine clearance for every 1ml decrease (RR = 0.95, 95% CI: 0.93–0.96) [51], hyperfiltration at baseline (HR = 4.1, 95% CI: 2.3–7.1) [52], higher serum creatine at baseline (OR = 49.8, 95% CI: 79–311.92) [41], and the presence of CKD.

### Publication bias

**Fig 7:**
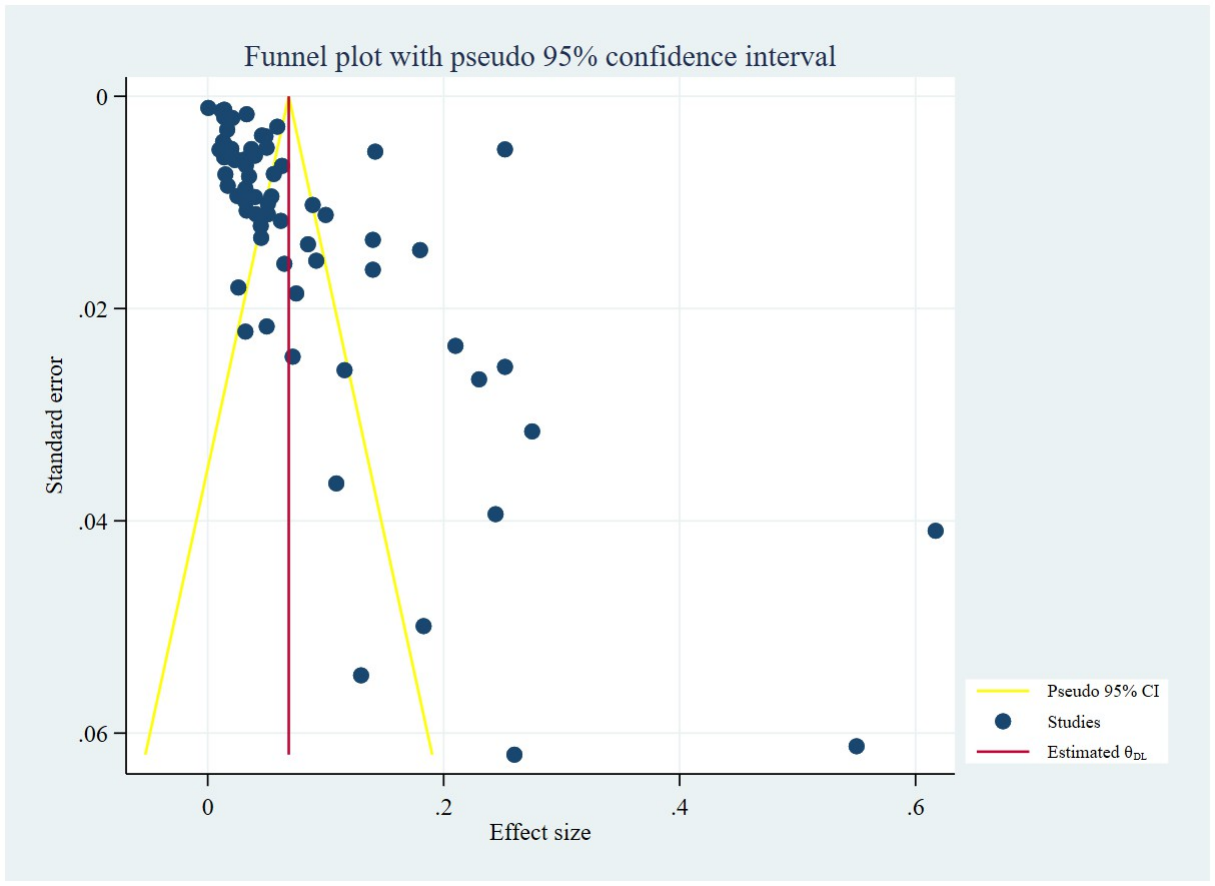
Funnel plot.

Included studies showed publication bias demonstrated by a funnel plot (Fig 7). We confirmed the significance of publication bias using egger’s statistical test (P <0.00001).

**Fig 8:**
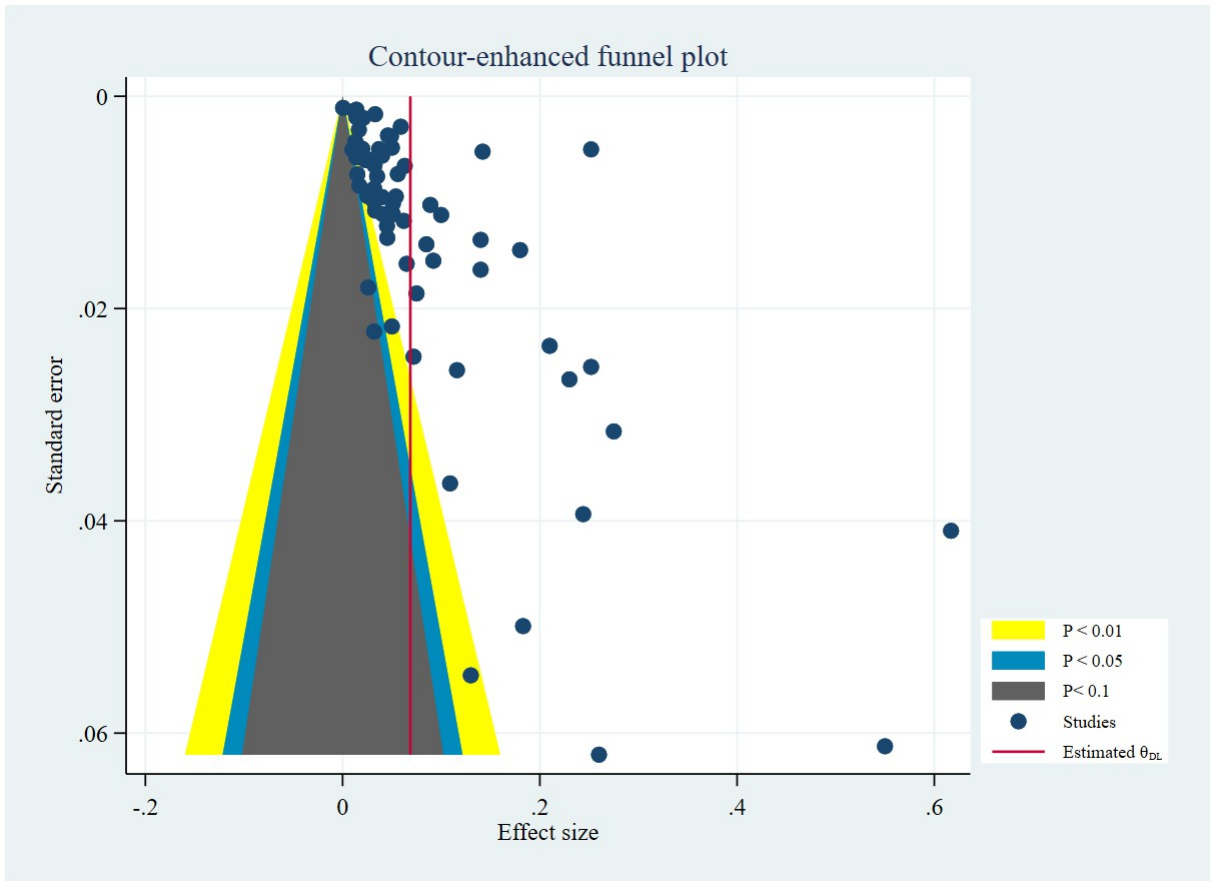
Contour enhanced funnel plot.

The contour enhanced funnel plot also showed publication bias (Fig 8).

**Fig 9:**
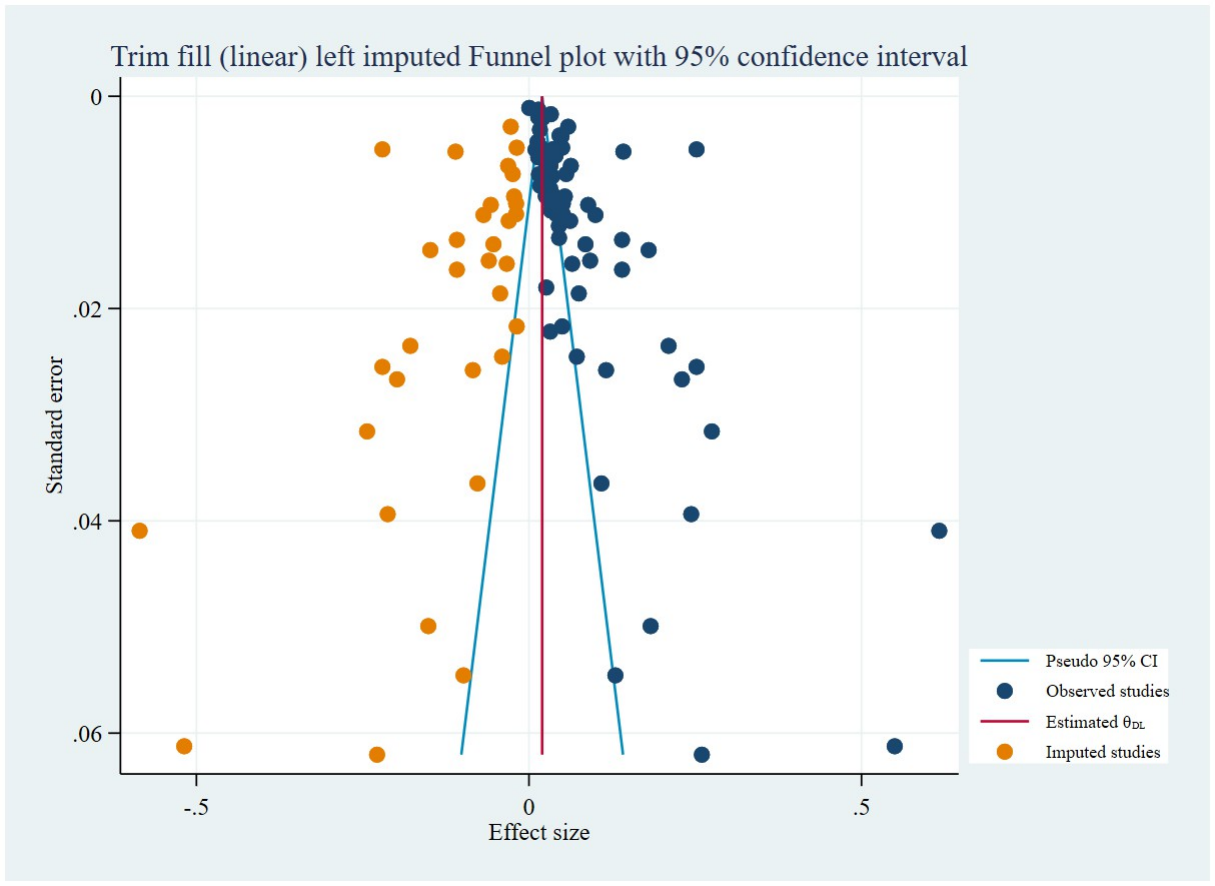
Funnel plot using nonparametric trim-and-fill analysis of publication bias.

To minimize the impact of publication bias on the pooled prevalence estimate of CKD, we performed a trim and fill analysis. We used a linear estimator with a fixed effect model to show imputed studies on left, followed by a random effects model to estimate the pooled prevalence of CKD using the trim and fill method. In this analysis, we found thirty-three imputed studies on left, and 2% (95% CI: 1–3%) overall pooled prevalence of CKD using the trim and fill method (Fig 9).

### Risk of bias of included studies

Risk of bias was assessed for eligible studies based on Hoy et al., 2012 risk assessment tool and the results have shown in S4 Table.

## Discussion

We conducted the present review to address the data gap regarding the pooled prevalence of CKD among PLWHIV receiving TDF based regimens across the world. In this review, we found a 7% (95% CI: 6–8%) pooled prevalence of CKD. The current review revealed that the prevalence of CKD among PLWHIV varied both within and between regions. The highest prevalence (11.7% [95% CI:8.4–15]) was observed in Sub-Sahara Africa region, while it was lower (3% [95% CI:2–4.1]) in other regions (studies conducted in more than one region). Additionally, the highest prevalence (23% [95% CI: 18–28%]) was found in Tanzania, whereas China showed the lowest burden of pooled prevalence of CKD (1% [95% CI: 0–2%]).

The present review included studies that used different eGFR estimation methods, and CKD diagnostic criteria with respect to time. Subgroup analysis found that the pooled prevalence of CKD based on CG, MDRD, and MDRD without race factor was significantly different from the overall pooled prevalence of CKD. Included studies used different methods, such as on the spot CKD diagnosis without baseline data, or with normal baseline data, or a decline to <50 or 60ml/min at two time points separated by at least 3 months for CKD confirmation. Subgroup analysis found significant difference among these groups.

Moreover, we found that the pooled CKD prevalence in LMIC, studies conducted in more than one income level, prospective cohort studies, and clinical trials significantly differed from the overall prevalence of CKD. The variation within and between regions may be related to differences in baseline study characteristics (comorbidity, comedication, renal function, WHO clinical stage of HIV/AIDS), duration of TDF exposure, eGFR estimation methods, income level, and study design [52, 56, 77, 86].

The interpretation of CKD in the present meta-analysis requires caution due to the high heterogeneity between studies. We performed sensitivity analysis by removing studies with potential outlier data, but heterogeneity and publication bias remained high, indicating unexplained heterogeneity.

The findings of this systematic review and meta-analysis are crucial for enhancing the quality of HIV care by providing a global picture of CKD in TDF based regimens, and suggesting strategies to prevent TDF related renal adverse effects. Furthermore, this review also has clinical importance in triggering healthcare policy makers worldwide to design strategies to optimize HIV care. Hence, optimizing TDF based regimens, and early CKD identification could help reduce further complication and mortality [93]. Our review finding suggests a re-evaluation of TDF based regimens in HIV care, which is in line with a systematic review summarizing nephrotoxicity in TDF based regimens across the globe [94]. Moreover, a scoping review supports our concern, highlighting a high burden of renal toxicity in TDF based regimens, and advocating for body weight or body mass index-based dosing [93]. A systematic review by Cooper RD et al. [10], and Mitsi TJ et al. [11], revealed a significant renal function decline in TDF based regimen, with a conclusion that such decline has modest clinical effect or was not enough to contradict its use. They recommend to consider consumer factors, and regular monitoring of renal function in TDF use [10, 11].

The mechanism of TDF induced nephrotoxicity is not well understood. However, the potential mechanism by which TDF causes kidney damage involves inhibition of mitochondrial DNA polymerase gamma [95]. Increased entry and decreased efflux of tenofovir by transport proteins in the renal tubule increase tenofovir induced renal toxicity [96].

Despite literature, and guidelines recommending the identification of patient factors, and regular monitoring of renal function in routine care, TDF initiation and use often occur without laboratory monitoring in low-income countries. In the present meta-analysis, we found significant association between being female, and CKD. This finding is in line with the evidences obtained from individual studies [50, 52, 56].

Moreover, CD4 count less than 200 copies/ml showed statistically significant association with CKD. These pooled effect supports the results from studies [50, 52, 56]. Studies of the present review showed factors associated with CKD, but we did not show their pooled effect due to either using different predictor category or statistical predictor models. These studies revealed that WHO stage [51, 86], cancer [77], HIV RNA viral load [31], blood pressure [19], SCr [41], eGFR [52], diabetes mellitus [56], ritonavir boosted protease inhibitor use [53], prior ART exposure [46], and TDF exposure duration [41, 86] have significant association with CKD in TDF based regimen. Health care providers should be vigilant to assess the risk of renal impairment in PLWHIV taking TDF. This review recommends monitoring of renal function before and during TDF use. In addition, it warrants the consideration gender at birth, and low CD4 count in routine health care practice to prevent CKD. Tenofovir alafenamide (TAF) is another prodrug formulation of tenofovir has better renal safety (but still has a concern) and equivalent efficacy compared to TDF [97, 98]. This safety difference was reported in reviews where TAF, and TDF have unique safety when they are used with pharmacokinetics boosters, but such difference was not seen when unboosted TAF compared to unboosted TDF [97, 98]. Evidence showed that using TDF has beneficial effect regarding lipid profiles [70], whereas TAF increases them [98]. Still guidelines consider both formulations as components of preferred regimens. High level evidences are demanded regarding overall safety, cost, and access of TDF versus TAF to modify clinical guidelines across the globe [98]. We recommend a large-scale post market study of TDF based regimen that help health policymakers to provide evidence-based decision.

This finding indicates high public health burden of CKD. Our finding has clinical implication that safety of TDF in HIV care where there is no regular baseline, and following up renal function monitoring is questionable, which is supported by other literature [94]. Considering this, and its high resource demanding management: we recommend to have a consensus on eGFR estimation equation to assess CKD in research, and health care practice as our review showed using different equations result in different results. The safety of TDF should be re-evaluated with high level evidences. In addition, health life style practice along with regular renal function monitoring has to be integrated with routine HIV care to prevent CKD.

Our review is the first to show the pooled estimate of CKD prevalence worldwide. However, it has limitations such as the CKD definition was not considering proteinuria or albuminuria; it was defined using only eGFR. Studies that estimated CKD on the spot were included, which may lead to under or over estimation of the pooled effect. In addition, studies did not give TDF with NNRTI, and PI/r specific data, so we did not show the pooled estimate of CKD in such subcategory. The included studies have high heterogeneity, and publication bias. we only included studies written in English. Further, we did non parametric trim and fill method analysis to minimize the impact of publication bias on the overall prevalence of CKD. Following trim and fill analysis, we found the pooled prevalence of CKD corrected for publication bias, which was 2% (95% CI: 1–3%).

## Conclusion

The present systematic review found a considerably high prevalence of CKD among HIV patients receiving TDF based regimens. A CD4 count of less than 200 copies per ml, and being female were significant predictor of CKD. Thus, we recommend regular renal function monitoring for PLWHIV receiving TDF. especially those with low CD4 counts, and females, to prevent, identify and manage CKD.

## Supporting information

S1 Table PRISMA 2020 checklist. (DOCX)

S2 Table Search strategy. (DOCX)

S3 Table Methodological quality assessment score of included studies. (DOCX)

S4 Table The risk of bias assessment tool results for the included studies. (DOCX)

S5 Table All accessed studies in literature search. (XLSX)

S6 Table Data extracted from included studies. (XLSX)

## Abbreviations

CG: Cockcroft-Gault
CKD: Chronic Kidney Disease
CKD-EPI: Chronic Kidney Disease-Epidemiology
eGFR: estimated Glomerular Filtration Rate
HIC: High Income Country
JSE: Japanese Society Equation
LIC: Low Income Country
LMIC: Lower Middle-Income Country
LUMIC: Lower Middle-Income Country
MDRD: Modification of Diet in Chronic Kidney Disease
PLWHIV: People Living With HIV
TDF: Tenofovir Disoproxil Fumarate
WHO: World Health Organization
