# Supplementary material for: Chronic kidney disease among people living with HIV on TDF based regimen: A systematic review and meta-analysis
Source: PLoS One. 2025 Feb 6;20(2):e0318068. doi: 10.1371/journal.pone.0318068 (PMC11801554; doi:10.1371/journal.pone.0318068)
Supplement: S2 Table — (DOCX) [file pone.0318068.s002.docx]

S2 Table. search strategy

| Database | Search query | Records retrieved |
| --- | --- | --- |
| EMBASE | ((('kidney failure'/exp OR 'kidney failure' OR 'renal impairment'/exp OR 'renal impairment' OR 'renal failure'/exp OR 'renal failure' OR 'renal insufficiency'/exp OR 'renal insufficiency') AND ('tenofovir'/exp OR 'tenofovir') OR 'tenofovir disoproxil fumarate'/exp OR 'tenofovir disoproxil fumarate') AND ('hiv'/exp OR 'hiv') OR 'aids'/exp OR 'aids' OR 'acquired immunodeficiency syndrome'/exp OR 'acquired immunodeficiency syndrome') AND [article in press]/lim AND [humans]/lim AND [english]/lim AND [medline]/lim AND [embase]/lim | 220 |
| Web of science | (((((((((((((TS=(chronic renal failure )) OR TS=(kidney failure)) OR TS=(renal impairment )) OR TS=(Kidney impairment )) OR TS=(End stage renal failure )) OR TS=(ESRD)) OR TS=(Renal  insufficiently )) AND TS=(Tenofovir)) OR TS=(tenovofir disoproxil fumarate)) OR TS=(TDF)) AND TS=(HIV)) OR TS=(human immunodeficiency virus)) OR TS=(acquired immunodeficiency syndrome)) OR TS=(AIDS) | 70 |
| CINHAL | (((((((((((((((((((((TI "Chronic Kidney Failure" OR AB "Chronic Kidney Failure")) OR ((TI "Chronic Renal Failure" OR AB "Chronic Renal Failure"))) OR ((TI "Chronic kidney disease" OR AB "Chronic kidney disease"))) OR ((TI "End-Stage Kidney Disease" OR AB "End-Stage Kidney Disease"))) OR ((TI "End-Stage Renal Disease" OR AB "End-Stage Renal Disease"))) OR ((TI "End-Stage Renal Failure" OR AB "End-Stage Renal Failure"))) OR ((TI ESRD OR AB ESRD))) OR ((TI "Renal Insufficiency" OR AB "Renal Insufficiency"))) OR ((TI "Renal impairment" OR AB "Renal impairment"))) OR ((TI "Kidney impairment" OR AB "Kidney impairment"))) OR ((TI "Renal failure" OR AB "Renal failure"))) OR ((TI "Kidney failure" OR AB "Kidney failure"))) OR ((TI "Renal dysfunction" OR AB "Renal dysfunction"))) OR ((TI "Kidney dysfunction" OR AB "Kidney dysfunction"))) AND ((TI Tenofovir OR AB Tenofovir))) OR ((TI "Tenofovir Disoproxil Fumarate" OR AB "Tenofovir Disoproxil Fumarate"))) OR ((TI TDF OR AB TDF))) AND ((TI HIV OR AB HIV))) OR ((TI "human immunodeficiency virus" OR AB "human immunodeficiency virus"))) OR ((TI AIDS OR AB AIDS))) OR ((TI "acquired immunodeficiency syndrome" OR AB "acquired immunodeficiency syndrome")) | 340 |
| Scopus | "Chronic Kidney Failure" OR "Chronic Renal Failure" OR "Chronic kidney disease" OR "End-Stage Kidney Disease" OR "End-Stage Renal Disease" OR "End-Stage Renal Failure" OR esrd OR "Renal Insufficiency" OR "Renal impairment" OR "Kidney impairment" OR "Renal failure" OR "Kidney failure" OR "Renal dysfunction" OR "Kidney dysfunction" AND tenofovir OR "Tenofovir Disoproxil Fumarate" OR tdf AND hiv OR hiv OR "human immunodeficiency virus" OR aids OR "acquired immunodeficiency syndrome." | 316 |
| PubMed | (("Chronic Kidney Failure" OR "Chronic Renal Failure" OR "Chronic kidney disease" OR "End-Stage Kidney Disease" OR "End-Stage Renal Disease" OR "End-Stage Renal Failure" OR "ESRD" OR "Renal Insufficiency" OR "Renal impairment" OR "Kidney impairment" OR "Renal failure" OR "Kidney failure" OR "Renal dysfunction" OR "Kidney dysfunction") AND ("Tenofovir" OR "Tenofovir Disoproxil Fumarate" OR "TDF")) AND ("HIV" OR "hiv" OR "human immunodeficiency virus" OR "AIDS " OR "acquired immunodeficiency syndrome") | 542 |
|  | "Chronic Kidney Failure" OR "Chronic Renal Failure" OR "Chronic kidney disease" OR "End-Stage Kidney Disease" OR "End-Stage Renal Disease" OR "End-Stage Renal Failure" OR "ESRD" OR "Renal Insufficiency" OR "Renal impairment" OR "Kidney impairment" OR "Renal failure" OR "Kidney failure" OR "Renal dysfunction" OR "Kidney dysfunction" | 3,02,302 |
|  | "Tenofovir" OR "Tenofovir Disoproxil Fumarate" OR "TDF" | 11,179 |
|  | "HIV" OR "hiv" OR "human immunodeficiency virus" OR "AIDS " OR "acquired immunodeficiency syndrome" | 5,35,771 |
