# Supplementary material for: Chronic kidney disease among people living with HIV on TDF based regimen: A systematic review and meta-analysis
Source: PLoS One. 2025 Feb 6;20(2):e0318068. doi: 10.1371/journal.pone.0318068 (PMC11801554; doi:10.1371/journal.pone.0318068)
Supplement: S3 Table — (DOCX) [file pone.0318068.s003.docx]

**Methodological quality assessment**

**S3.** Methodological quality assessment score of included studies

**Table 1**: Methodological quality assessment of included cross sectional studies using modified Newcastle - Ottawa Scale (NOS)

| **Author** | **Criteria** | | | | | | | |  |
| --- | --- | --- | --- | --- | --- | --- | --- | --- | --- |
|  | **Selection** | | | | **Comparability** | | **Outcome** | |  |
|  | **Representativeness of the sample** | **Sample size** | **Non –respondents** | **Ascertainment of exposure/risk factor** | **The study controls for the most important factor** | **The study control for any additional factor** | **Assessment of the outcome** | **Statistical test** | **Quality score** |
| Belete AM and Yazie TS, 2021 | 1 | 0 | 1 | 1 | 1 | 1 | 2 | 1 | 8 |
| Fritzsche C et al.,2017 | 1 | 0 | 1 | 1 | 1 | 1 | 0 | 1 | 6 |
| Chadwick DR et al., 2015 | 1 | 0 | 1 | 1 | 1 | 1 | 0 | 1 | 6 |
| Nyende L et al., 2020 | 1 | 1 | 1 | 1 | 1 | 1 | 2 | 0 | 8 |
| Okpa HO et al., 2019 | 1 | 0 | 1 | 1 | 1 | 1 | 2 | 0 | 7 |
| Jotwani V et al., 2016 | 0 | 0 | 1 | 1 | 1 | 1 | 2 | 1 | 7 |
| Nishijima T et al., 2017 | 1 | **0** | **1** | **1** | **1** | **1** | 0 | **1** | **6** |
| Obiri-Yeboah D et al., 2018 | 1 | **1** | **1** | **1** | **1** | **1** | 0 | **0** | **6** |
| Calza L et al., 2014 | 1 | **0** | **1** | **1** | **1** | **1** | 2 | **0** | **8** |
| Likanonsakul S et al.,2016 | 1 | **0** | **1** | **1** | **1** | **1** | 2 | **0** | **7** |
| Ahmed E et al., 2020 | 1 | **1** | **1** | **1** | **1** | **1** | 2 | **0** | **8** |
| Juega-Mariño J et al., 2017 | 1 | **0** | **1** | **1** | **1** | **1** | 2 | **0** | **7** |
| Mwemezi O et al., 2020 | 1 | **1** | **1** | **1** | **1** | **1** | 2 | **0** | **8** |
| Reynes J et al., 2013 | 1 | **0** | **1** | **0** | **1** | **1** | 2 | **0** | **6** |
| Hoang C et al., 2020 | 1 | **1** | **1** | **1** | **1** | **1** | 2 | **0** | **8** |
| Crum-Cianflone N et al., 2010 | 1 | **0** | **1** | **1** | **1** | **1** | 2 | **0** | **7** |

*Note: from each item account point. (Accept the study for each study design based on total score of ≥50%)*

Selection: (Maximum 5 stars)
1) Representativeness of the sample: a) Truly representative of the average in the target population. * (all subjects or random sampling) .b) Somewhat representative of the average in the target population. * (nonrandom sampling) .c) Selected group of users.d) No description of the sampling strategy.
2) Sample size:a) Justified and satisfactory. *.b) Not justified.
3) Non-respondents: a) Comparability between respondents and non-respondents characteristics is
established, and the response rate is satisfactory. * .b) The response rate is unsatisfactory, or the comparability between respondents
and non-respondents is unsatisfactory. c) No description of the response rate or the characteristics of the responders and
the non-responders.
4) Ascertainment of the exposure (risk factor): a) validated measurement tool. ** .b) Non-validated measurement tool, but the tool is available or described.* c) No description of the measurement tool.
Comparability: (Maximum 2 stars)
1) The subjects in different outcome groups are comparable, based on the study design or analysis. Confounding factors are controlled. a) The study controls for the most important factor (select one). * b) The study control for any additional factor. *
Outcome: (Maximum 3 stars)
1) Assessment of the outcome: a) Independent blind assessment. **,b) Record linkage. **,c) Self report. *,d) No description.
2) Statistical test:a) The statistical test used to analyze the data is clearly described and appropriate, and the measurement of the association is presented, including confidence intervals and the probability level (p value). *,b) The statistical test is not appropriate, not described or incomplete

**Table 2:** Methodological quality assessment of included cohort studies using modified Newcastle - Ottawa Scale (NOS)

| **Author** |  | **Criteria** | | | | | | | | |  |
| --- | --- | --- | --- | --- | --- | --- | --- | --- | --- | --- | --- |
|  |  | **Selection** | | | | **Comparability** | | **Outcome** | | |  |
|  | **Study design** | **Representative exposed** | **Selection of non-cohort** | **Ascertainment of exposure** | **Demonstration no outcome at start** | **Control most important factor** | **Control additional important factor** | **Assessment of outcome** | **Follow up long enough for outcome** | **Follow up adequate for cohort** | **Quality score** |
| Neary M et al., 2020 | ProCT | 1 | 1 | 1 | 1 | 1 | 1 | 1 | 1 | 1 | 9 |
| Bock P et al., 2019 | RetCT | 1 | 1 | 1 | 1 | 1 | 1 | 1 | 0 | 1 | 8 |
| Debeb SG et al., 2021 | RetCT | 1 | 1 | 1 | 1 | 1 | 1 | 1 | 1 | 1 | 9 |
| Chikwapulo B et al., 2018 | RetCT | 1 | 1 | 1 | 1 | 1 | 1 | 1 | 1 | 1 | 9 |
| Yazie TS et al., 2019 | ProCT | 1 | 1 | 1 | 1 | 1 | 1 | 1 | 1 | 1 | 9 |
| Zachor H et al., 2016 | RetCT | 1 | 1 | 1 | 1 | 1 | 1 | 1 | 1 | 1 | 8 |
| Ojen BV et al., 2018 | RetCT | 1 | 1 | 1 | 1 | 1 | 1 | 1 | 1 | 1 | 9 |
| Nartey ET et al., 2019 | RetCT | 1 | 1 | 1 | 1 | 1 | 1 | 1 | 1 | 1 | 9 |
| Kalemeera F et al., 2020 | RetCT | 1 | 1 | 1 | 1 | 1 | 1 | 1 | 1 | 1 | 9 |
| Pujari SN., 2014 | RetCT | 1 | 1 | 1 | 1 | 1 | 1 | 1 | 1 | 1 | 9 |
| Visuthrankul J et al., 2021 | RetCT | 1 | 1 | 1 | 1 | 1 | 1 | 1 | 1 | 1 | 9 |
| Nishijima T et al., 2014 | ProCT | 1 | 1 | 1 | 1 | 1 | 1 | 1 | 1 | 1 | 9 |
| Kyaw NTT et al., 2015 | RetCT | 1 | 1 | 1 | 1 | 1 | 1 | 1 | 0 | 1 | 8 |
| O'Donnel EP et al., 2011 | RetCT | 1 | 1 | 1 | 1 | 1 | 1 | 1 | 1 | 1 | 9 |
| Nishijima T et al., 2011 | RetCT | 1 | 1 | 1 | 1 | 1 | 1 | 1 | 1 | 0 | 8 |
| Woolnough EL et al., 2018 | RetCT | 1 | 1 | 1 | 1 | 1 | 1 | 1 | 1 | 1 | 9 |
| Hsu R et al., 2020 | RetCT | 1 | 1 | 1 | 1 | 1 | 1 | 1 | 1 | 1 | 9 |
| Lapadula G et al., 2016 | ProCT | 1 | 1 | 1 | 1 | 1 | 1 | 1 | 1 | 1 | 9 |
| Morlat P et al., 2013 | ProCT | 1 | 1 | 1 | 1 | 1 | 1 | 1 | 1 | 1 | 9 |
| Nishijima T et al., 2016 | ProCT | 1 | 1 | 1 | 1 | 1 | 1 | 1 | 1 | 1 | 9 |
| Kim JH et al., 2022 | ProCT | 0 | 1 | 1 | 1 | 1 | 1 | 1 | 1 | 1 | 8 |
| Young et al., 2007 | ProCT | 1 | 1 | 1 | 1 | 1 | 1 | 1 | 1 | 1 | 9 |
| Feng L et al., 2022 | ProCT | 1 | 1 | 1 | 1 | 1 | 1 | 1 | 1 | 1 | 9 |
| Sutton SS et al., 2020 | RetCT | 1 | 1 | 1 | 1 | 1 | 1 | 1 | 1 | 1 | 9 |
| Cheung J et al., 2018 | ProCT | 1 | 1 | 1 | 1 | 1 | 1 | 1 | 1 | 1 | 9 |
| Tan LKK et al., 2009 | RetCT | 1 | 1 | 1 | 1 | 1 | 1 | 1 | 1 | 1 | 9 |
| Milazzo L et al., 2016 | RetCT | 1 | 1 | 1 | 1 | 1 | 1 | 1 | 1 | 1 | 9 |
| Quesada PR et al., 2015 | ProCT | 1 | 1 | 1 | 1 | 1 | 1 | 1 | 1 | 1 | 9 |
| Low JZ et al., 2018 | RetCT | 1 | 1 | 1 | 1 | 1 | 1 | 1 | 1 | 1 | 9 |
| Chabala FW et al., 2021 | ProCT | 1 | 1 | 1 | 1 | 1 | 1 | 1 | 1 | 1 | 9 |
| Flandre P et al.,2016 | ProCT | 1 | 1 | 1 | 1 | 1 | 1 | 1 | 1 | 1 | 9 |
| Suzuki S et al.,2017 | RetCT | 1 | 1 | 1 | 1 | 1 | 1 | 1 | 1 | 1 | 9 |
| Domingo P et al.,2019 | RetCT | 1 | 1 | 1 | 1 | 1 | 1 | 1 | 1 | 1 | 9 |
| Lee KH et al.,2017 | RetCT | 1 | 1 | 1 | 1 | 1 | 1 | 1 | 1 | 1 | 9 |
| Paengsai N et al.,2022 | RetCT | 1 | 1 | 1 | 1 | 1 | 1 | 1 | 1 | 1 | 9 |
| Ando M et al., 2011 | ProCT | 1 | 1 | 1 | 1 | 1 | 1 | 1 | 1 | 1 | 9 |
| Chua AC et al., 2012 | RetCT | 1 | 1 | 1 | 1 | 1 | 1 | 1 | 1 | 1 | 9 |
| Nishijima T et al., 2015 | RetCT | 1 | 1 | 1 | 1 | 1 | 1 | 1 | 1 | 1 | 9 |
| Huang Y et al., 2017 | RetCT | 1 | 1 | 1 | 1 | 1 | 1 | 1 | 1 | 1 | 9 |
| Calza L et al.,2013 | RetCT | 1 | 1 | 1 | 1 | 1 | 1 | 1 | 1 | 1 | 9 |
| Yang J et al., 2019 | RetCT | 1 | 1 | 1 | 1 | 1 | 1 | 1 | 1 | 1 | 9 |
| Monteagudo-Chu et al., 2012 | RetCT | 1 | 1 | 1 | 1 | 1 | 1 | 1 | 1 | 1 | 9 |
| Medland NA et al., 2017 | RetCT | 1 | 1 | 0 | 1 | 1 | 1 | 1 | 1 | 1 | 8 |
| Suppadungsuk S et al., 2022 | RetCT | 1 | 1 | 1 | 1 | 1 | 1 | 1 | 1 | 1 | 9 |
| Pedrol E et al., 2015 | RetCT | 1 | 1 | 1 | 1 | 1 | 1 | 1 | 1 | 1 | 9 |
| Kalemeera F et al., 2023 | RetCT | 1 | 1 | 1 | 1 | 1 | 1 | 1 | 1 | 1 | 9 |
| Joshi et al., 2019 | RetCT | 1 | 1 | 1 | 1 | 1 | 1 | 1 | 1 | 1 | 9 |
| Mocroft A et al., 2015 | ProCT | 1 | 1 | 1 | 1 | 1 | 1 | 1 | 1 | 1 | 9 |
| Liu F et al., 2021 | RetCT | 1 | 1 | 1 | 1 | 1 | 1 | 1 | 1 | 1 | 9 |
| Campbell LJ et al.,2009 | RetCT | **1** | **1** | **1** | **1** | **1** | 1 | **1** | **1** | 1 | 9 |

**Table 3:Summary of** methodological quality assessment of included randomized controlled trials studies using Revised Cochrane risk-of-bias tool for randomized trials (RoB 2)

| **Author** | **Criteria** | | | | |  |
| --- | --- | --- | --- | --- | --- | --- |
|  | **Domain 1: Risk of bias arising from the randomization process** | **Domain 2: Risk of bias due to deviations from the intended interventions (effect of assignment to intervention)** | **Domain 3: Missing outcome data** | **Domain 4: Risk of bias in measurement of the outcome** | **Domain 5: Risk of bias in selection of the reported result** | **Overall Risk judgement** |
| Cournil A et al. 2017 | Low | Low | Low | Low | Low | Low |
| Mwafongo A et al., 2015 | Some concerns | Low | Low | Some concerns | Low | Some concerns |
| Chan A et al., 2019 | High | Low | Low | Low | Low | High |

**Table 3.1**. Assessment results of the level of risk of bias for each domain and answers for each signalling questions within each domain using Cochrane risk-of-bias tool for randomized trials.

Table 3.1.1. Results of risk of bias for study by Cournil A et al., 2017.

Revised Cochrane risk-of-bias tool for randomized trials (RoB 2)

TEMPLATE FOR COMPLETION

Edited by Julian PT Higgins, Jelena Savović, Matthew J Page, Jonathan AC Sterne
on behalf of the RoB2 Development Group

**Version of 22 August 2019**

The development of the RoB 2 tool was supported by the MRC Network of Hubs for Trials Methodology Research (MR/L004933/2- N61), with the support of the host MRC ConDuCT-II Hub (Collaboration and innovation for Difficult and Complex randomised controlled Trials In Invasive procedures - MR/K025643/1), by MRC research grant MR/M025209/1, and by a grant from The Cochrane Collaboration.


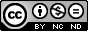


This work is licensed under a [Creative Commons Attribution-NonCommercial-NoDerivatives 4.0 International License](http://creativecommons.org/licenses/by-nc-nd/4.0/).

| **Study details**   \| **Reference** \| Cournil A, Hema A, Eymard-Duvernay S, Ciaffi L, Badiou S, Kabore FN, *et al*. Evolution of renal function in African patients initiating second-line antiretroviral treatment: findings from the ANRS 12169 2LADY trial. Antiviral Therapy. 2017 Apr;22(3):195-203. \| \| --- \| --- \|   **Study design**   \| X \| Individually-randomized parallel-group trial \| \| --- \| --- \| \| □ \| Cluster-randomized parallel-group trial \| \| □ \| Individually randomized cross-over (or other matched) trial \|   **For the purposes of this assessment, the interventions being compared are defined as**   \| Experimental: \| TDF based regimen \| Comparator: \| Abacavir based regimen \| \| --- \| --- \| --- \| --- \|  \| **Specify which outcome is being assessed for risk of bias** \| CKD \| \| --- \| --- \|  \| **Specify the numerical result being assessed.** In case of multiple alternative analyses being presented, specify the numeric result (e.g. RR = 1.52 (95% CI 0.83 to 2.77) and/or a reference (e.g. to a table, figure or paragraph) that uniquely defines the result being assessed. \| eGFR <50 or 60ml/min \| \| --- \| --- \|   **Is the review team’s aim for this result…?**   \| 🞫 \| to assess the effect of *assignment to intervention* (the ‘intention-to-treat’ effect) \| \| --- \| --- \| \| □ \| to assess the effect of *adhering to intervention* (the ‘per-protocol’ effect) \|   **If the aim is to assess the effect of *adhering to intervention***, select the deviations from intended intervention that should be addressed (at least one must be checked):  □ occurrence of non-protocol interventions  □ failures in implementing the intervention that could have affected the outcome  □ non-adherence to their assigned intervention by trial participants  **Which of the following sources were obtained to help inform the risk-of-bias assessment? (tick as many as apply)**  🞫 Journal article(s) with results of the trial  □ Trial protocol  □ Statistical analysis plan (SAP)  □ Non-commercial trial registry record (e.g. ClinicalTrials.gov record)  □ Company-owned trial registry record (e.g. GSK Clinical Study Register record)  □ “Grey literature” (e.g. unpublished thesis)  □ Conference abstract(s) about the trial  □ Regulatory document (e.g. Clinical Study Report, Drug Approval Package)  □ Research ethics application  □ Grant database summary (e.g. NIH RePORTER or Research Councils UK Gateway to Research)  □ Personal communication with trialist  □ Personal communication with the sponsor |
| --- | --- | --- | --- | --- | --- | --- | --- | --- | --- | --- | --- | --- | --- | --- | --- | --- | --- | --- | --- | --- |

Risk of bias assessment

Responses underlined in green are potential markers for low risk of bias, and responses in red are potential markers for a risk of bias. Where questions relate only to sign posts to other questions, no formatting is used.

**Domain 1: Risk of bias arising from the randomization process**

| **Signalling questions** | **Comments** | **Response options** |
| --- | --- | --- |
| **1.1 Was the allocation sequence random?** | 1:1:1 randomization was stated in the article.  No information provided | Y |
| **1.2 Was the allocation sequence concealed until participants were enrolled and assigned to interventions?** |  | PY |
| **1.3 Did baseline differences between intervention groups suggest a problem with the randomization process?** | Baseline characteristics were balanced between treatment arms. | N |
| **Risk-of-bias judgement** |  | Low risk |
| Optional: What is the predicted direction of bias arising from the randomization process? |  | NA |

Domain 2: Risk of bias due to deviations from the intended interventions (*effect of assignment to intervention*)

| **Signalling questions** | **Comments** | **Response options** |
| --- | --- | --- |
| **2.1. Were participants aware of their assigned intervention during the trial?** | Oen label randomized trial | Y |
| **2.2. Were carers and people delivering the interventions aware of participants' assigned intervention during the trial?** |  | Y |
| **2.3. If Y/PY/NI to 2.1 or 2.2: Were there deviations from the intended intervention that arose because of the trial context?** | No | N |
| **2.4 If Y/PY to 2.3: Were these deviations likely to have affected the outcome?** |  | NA |
| **2.5. If Y/PY/NI to 2.4: Were these deviations from intended intervention balanced between groups?** |  | NA |
| **2.6 Was an appropriate analysis used to estimate the effect of assignment to intervention?** | Yes | Y |
| **2.7 If N/PN/NI to 2.6: Was there potential for a substantial impact (on the result) of the failure to analyse participants in the group to which they were randomized?** |  | NA |
| **Risk-of-bias judgement** |  | Low |
| Optional: What is the predicted direction of bias due to deviations from intended interventions? |  | NA |

Domain 2: Risk of bias due to deviations from the intended interventions (*effect of adhering to intervention*)

| **Signalling questions** | **Comments** | **Response options** |
| --- | --- | --- |
| **2.1. Were participants aware of their assigned intervention during the trial?** | Yes  Yes | Y |
| **2.2. Were carers and people delivering the interventions aware of participants' assigned intervention during the trial?** |  | Y |
| **2.3. [If applicable:] If Y/PY/NI to 2.1 or 2.2: Were important non-protocol interventions balanced across intervention groups?** | Yes | Y |
| **2.4. [If applicable:] Were there failures in implementing the intervention that could have affected the outcome?** | The groups parameters were made balanced | NA |
| **2.5. [If applicable:] Was there non-adherence to the assigned intervention regimen that could have affected participants’ outcomes?** | The groups parameters were made balanced | NA |
| **2.6. If N/PN/NI to 2.3, or Y/PY/NI to 2.4 or 2.5: Was an appropriate analysis used to estimate the effect of adhering to the intervention?** | The groups parameters were made balanced | NA |
| **Risk-of-bias judgement** |  | Low |
| Optional: What is the predicted direction of bias due to deviations from intended interventions? |  | NA |

Domain 3: Missing outcome data

| **Signalling questions** | **Comments** | **Response options** |
| --- | --- | --- |
| **3.1 Were data for this outcome available for all, or nearly all, participants randomized?** | >10% lost follow up | N |
| **3.2 If N/PN/NI to 3.1: Is there evidence that the result was not biased by missing outcome data?** | No information | N |
| **3.3 If N/PN to 3.2: Could missingness in the outcome depend on its true value?** | No  NA | N |
| **3.4 If Y/PY/NI to 3.3: Is it likely that missingness in the outcome depended on its true value?** |  | NA |
| **Risk-of-bias judgement** |  | Low |
| Optional: What is the predicted direction of bias due to missing outcome data? |  | Unpredictable |

Domain 4: Risk of bias in measurement of the outcome

| **Signalling questions** | **Comments** | **Response options** |
| --- | --- | --- |
| **4.1 Was the method of measuring the outcome inappropriate?** | CKD diagnosed by eGFR <60ml/min estimated by MDRD | N |
| **4.2 Could measurement or ascertainment of the outcome have differed between intervention groups?** | No | N |
| **4.3 If N/PN/NI to 4.1 and 4.2: Were outcome assessors aware of the intervention received by study participants?** | Probably yes as it is open label trial | Y |
| **4.4 If Y/PY/NI to 4.3: Could assessment of the outcome have been influenced by knowledge of intervention received?** | No information  NA | PN |
| **4.5 If Y/PY/NI to 4.4:** **Is it likely that assessment of the outcome was influenced by knowledge of intervention received?** |  | NA |
| **Risk-of-bias judgement** |  | Low |
| Optional: What is the predicted direction of bias in measurement of the outcome? |  | NA |

Domain 5: Risk of bias in selection of the reported result

| **Signalling questions** | **Comments** | **Response options** |
| --- | --- | --- |
| **5.1 Were the data that produced this result analysed in accordance with a pre-specified analysis plan that was finalized before unblinded outcome data were available for analysis?** | Yes | Y |
| **Is the numerical result being assessed likely to have been selected, on the basis of the results, from...** |  |  |
| **5.2. ... multiple eligible outcome measurements (e.g. scales, definitions, time points) within the outcome domain?** | No | N |
| **5.3 ... multiple eligible analyses of the data?** | No | N |
| **Risk-of-bias judgement** |  | Low |
| Optional: What is the predicted direction of bias due to selection of the reported result? |  | NA |

Overall risk of bias

| **Risk-of-bias judgement** | All domains have low risk | Low |
| --- | --- | --- |
| Optional: What is the overall predicted direction of bias for this outcome? |  | NA |


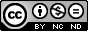


This work is licensed under a [Creative Commons Attribution-NonCommercial-NoDerivatives 4.0 International License](http://creativecommons.org/licenses/by-nc-nd/4.0/)

Table 3.1.2. Results of risk of bias for study by Mwafongo A et al., 2014.

Revised Cochrane risk-of-bias tool for randomized trials (RoB 2)

TEMPLATE FOR COMPLETION

Edited by Julian PT Higgins, Jelena Savović, Matthew J Page, Jonathan AC Sterne
on behalf of the RoB2 Development Group

**Version of 22 August 2019**

The development of the RoB 2 tool was supported by the MRC Network of Hubs for Trials Methodology Research (MR/L004933/2- N61), with the support of the host MRC ConDuCT-II Hub (Collaboration and innovation for Difficult and Complex randomised controlled Trials In Invasive procedures - MR/K025643/1), by MRC research grant MR/M025209/1, and by a grant from The Cochrane Collaboration.


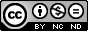


This work is licensed under a [Creative Commons Attribution-NonCommercial-NoDerivatives 4.0 International License](http://creativecommons.org/licenses/by-nc-nd/4.0/).

| **Study details**   \| **Reference** \| Mwafongo A, Nkanaunena K, Zheng Y, Hogg E, Samaneka W, Mulenga L, *et al*. Renal events among women treated with tenofovir/emtricitabine in combination with either lopinavir/ritonavir or nevirapine. Aids. 2014 May 15;28(8):1135-42. \| \| --- \| --- \|   **Study design**   \| X \| Individually-randomized parallel-group trial \| \| --- \| --- \| \| □ \| Cluster-randomized parallel-group trial \| \| □ \| Individually randomized cross-over (or other matched) trial \|   **For the purposes of this assessment, the interventions being compared are defined as**   \| Experimental: \| Ritonavir boosted lopinavir based TDF regimen \| Comparator: \| Nevirapine based TDF regimen \| \| --- \| --- \| --- \| --- \|  \| **Specify which outcome is being assessed for risk of bias** \| CKD \| \| --- \| --- \|  \| **Specify the numerical result being assessed.** In case of multiple alternative analyses being presented, specify the numeric result (e.g. RR = 1.52 (95% CI 0.83 to 2.77) and/or a reference (e.g. to a table, figure or paragraph) that uniquely defines the result being assessed. \| eGFR <50 or 60ml/min \| \| --- \| --- \|   **Is the review team’s aim for this result…?**   \| 🞫 \| to assess the effect of *assignment to intervention* (the ‘intention-to-treat’ effect) \| \| --- \| --- \| \| □ \| to assess the effect of *adhering to intervention* (the ‘per-protocol’ effect) \|   **If the aim is to assess the effect of *adhering to intervention***, select the deviations from intended intervention that should be addressed (at least one must be checked):  □ occurrence of non-protocol interventions  □ failures in implementing the intervention that could have affected the outcome  □ non-adherence to their assigned intervention by trial participants  **Which of the following sources were obtained to help inform the risk-of-bias assessment? (tick as many as apply)**  🞫 Journal article(s) with results of the trial  □ Trial protocol  □ Statistical analysis plan (SAP)  □ Non-commercial trial registry record (e.g. ClinicalTrials.gov record)  □ Company-owned trial registry record (e.g. GSK Clinical Study Register record)  □ “Grey literature” (e.g. unpublished thesis)  □ Conference abstract(s) about the trial  □ Regulatory document (e.g. Clinical Study Report, Drug Approval Package)  □ Research ethics application  □ Grant database summary (e.g. NIH RePORTER or Research Councils UK Gateway to Research)  □ Personal communication with trialist  □ Personal communication with the sponsor |
| --- | --- | --- | --- | --- | --- | --- | --- | --- | --- | --- | --- | --- | --- | --- | --- | --- | --- | --- | --- | --- |

Risk of bias assessment

Responses underlined in green are potential markers for low risk of bias, and responses in red are potential markers for a risk of bias. Where questions relate only to sign posts to other questions, no formatting is used.

**Domain 1: Risk of bias arising from the randomization process**

| **Signalling questions** | **Comments** | **Response options** |
| --- | --- | --- |
| **1.1 Was the allocation sequence random?** | Randomization was stated in the article.  No information provided | Y |
| **1.2 Was the allocation sequence concealed until participants were enrolled and assigned to interventions?** |  | NI |
| **1.3 Did baseline differences between intervention groups suggest a problem with the randomization process?** | Baseline characteristics were balanced between treatment arms. | N |
| **Risk-of-bias judgement** |  | Some concerns |
| Optional: What is the predicted direction of bias arising from the randomization process? |  | NA |

Domain 2: Risk of bias due to deviations from the intended interventions (*effect of assignment to intervention*)

| **Signalling questions** | **Comments** | **Response options** |
| --- | --- | --- |
| **2.1. Were participants aware of their assigned intervention during the trial?** | No information  No information | NI |
| **2.2. Were carers and people delivering the interventions aware of participants' assigned intervention during the trial?** |  | NI |
| **2.3. If Y/PY/NI to 2.1 or 2.2: Were there deviations from the intended intervention that arose because of the trial context?** | No | PN |
| **2.4 If Y/PY to 2.3: Were these deviations likely to have affected the outcome?** | NA | NA |
| **2.5. If Y/PY/NI to 2.4: Were these deviations from intended intervention balanced between groups?** | NA | NA |
| **2.6 Was an appropriate analysis used to estimate the effect of assignment to intervention?** | Yes | Y |
| **2.7 If N/PN/NI to 2.6: Was there potential for a substantial impact (on the result) of the failure to analyse participants in the group to which they were randomized?** | NA | NA |
| **Risk-of-bias judgement** |  | Low |
| Optional: What is the predicted direction of bias due to deviations from intended interventions? |  | NA |

Domain 2: Risk of bias due to deviations from the intended interventions (*effect of adhering to intervention*)

| **Signalling questions** | **Comments** | **Response options** |
| --- | --- | --- |
| **2.1. Were participants aware of their assigned intervention during the trial?** | No information  No information | NI |
| **2.2. Were carers and people delivering the interventions aware of participants' assigned intervention during the trial?** |  | NI |
| **2.3. [If applicable:] If Y/PY/NI to 2.1 or 2.2: Were important non-protocol interventions balanced across intervention groups?** | Yes | Y |
| **2.4. [If applicable:] Were there failures in implementing the intervention that could have affected the outcome?** | The groups parameters were made balanced | NA |
| **2.5. [If applicable:] Was there non-adherence to the assigned intervention regimen that could have affected participants’ outcomes?** | The groups parameters were made balanced | NA |
| **2.6. If N/PN/NI to 2.3, or Y/PY/NI to 2.4 or 2.5: Was an appropriate analysis used to estimate the effect of adhering to the intervention?** | The groups parameters were made balanced | NA |
| **Risk-of-bias judgement** |  | Low |
| Optional: What is the predicted direction of bias due to deviations from intended interventions? |  | NA |

Domain 3: Missing outcome data

| **Signalling questions** | **Comments** | **Response options** |
| --- | --- | --- |
| **3.1 Were data for this outcome available for all, or nearly all, participants randomized?** | <5% lost follow up | Y |
| **3.2 If N/PN/NI to 3.1: Is there evidence that the result was not biased by missing outcome data?** | NA | NA |
| **3.3 If N/PN to 3.2: Could missingness in the outcome depend on its true value?** | NA  NA | NA |
| **3.4 If Y/PY/NI to 3.3: Is it likely that missingness in the outcome depended on its true value?** |  | NA |
| **Risk-of-bias judgement** |  | Low |
| Optional: What is the predicted direction of bias due to missing outcome data? |  | NA |

Domain 4: Risk of bias in measurement of the outcome

| **Signalling questions** | **Comments** | **Response options** |
| --- | --- | --- |
| **4.1 Was the method of measuring the outcome inappropriate?** | CKD diagnosed by eGFR <60ml/min estimated by MDRD | N |
| **4.2 Could measurement or ascertainment of the outcome have differed between intervention groups?** | No | N |
| **4.3 If N/PN/NI to 4.1 and 4.2: Were outcome assessors aware of the intervention received by study participants?** | No information | NI |
| **4.4 If Y/PY/NI to 4.3: Could assessment of the outcome have been influenced by knowledge of intervention received?** | No information  No information | NI |
| **4.5 If Y/PY/NI to 4.4: Is it likely that assessment of the outcome was influenced by knowledge of intervention received?** |  | NI |
| **Risk-of-bias judgement** |  | Some concerns |
| Optional: What is the predicted direction of bias in measurement of the outcome? |  | Unpredictable |

Domain 5: Risk of bias in selection of the reported result

| **Signalling questions** | **Comments** | **Response options** |
| --- | --- | --- |
| **5.1 Were the data that produced this result analysed in accordance with a pre-specified analysis plan that was finalized before unblinded outcome data were available for analysis?** | Yes | Y |
| **Is the numerical result being assessed likely to have been selected, on the basis of the results, from...** |  |  |
| **5.2. ... multiple eligible outcome measurements (e.g. scales, definitions, time points) within the outcome domain?** | No | N |
| **5.3 ... multiple eligible analyses of the data?** | No | N |
| **Risk-of-bias judgement** |  | Low |
| Optional: What is the predicted direction of bias due to selection of the reported result? |  | NA |

Overall risk of bias

| **Risk-of-bias judgement** | Two domains have some concern risk results | Some concern |
| --- | --- | --- |
| Optional: What is the overall predicted direction of bias for this outcome? |  | Unpredictable |


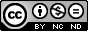


This work is licensed under a [Creative Commons Attribution-NonCommercial-NoDerivatives 4.0 International License](http://creativecommons.org/licenses/by-nc-nd/4.0/)

Table 3.1.3. Results of risk of bias for study by Chan A et al., 2019.

Revised Cochrane risk-of-bias tool for randomized trials (RoB 2)

TEMPLATE FOR COMPLETION

Edited by Julian PT Higgins, Jelena Savović, Matthew J Page, Jonathan AC Sterne
on behalf of the RoB2 Development Group

**Version of 22 August 2019**

The development of the RoB 2 tool was supported by the MRC Network of Hubs for Trials Methodology Research (MR/L004933/2- N61), with the support of the host MRC ConDuCT-II Hub (Collaboration and innovation for Difficult and Complex randomised controlled Trials In Invasive procedures - MR/K025643/1), by MRC research grant MR/M025209/1, and by a grant from The Cochrane Collaboration.


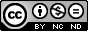


This work is licensed under a [Creative Commons Attribution-NonCommercial-NoDerivatives 4.0 International License](http://creativecommons.org/licenses/by-nc-nd/4.0/).

| **Study details**   \| **Reference** \| Chan A, Park L, Collins LF, Cooper C, Saag M, Dieterich D, *et al*. Correlation between tenofovir drug levels and the renal biomarkers RBP-4 and ß2M in the ION-4 study cohort. InOpen forum infectious diseases 2019 Jan (Vol. 6, No. 1, p. ofy273). US: Oxford University Press. \| \| --- \| --- \|   **Study design**   \| X \| Individually-randomized parallel-group trial \| \| --- \| --- \| \| □ \| Cluster-randomized parallel-group trial \| \| □ \| Individually randomized cross-over (or other matched) trial \|   **For the purposes of this assessment, the interventions being compared are defined as**   \| Experimental: \| TDF based regimen \| Comparator: \|  \| \| --- \| --- \| --- \| --- \|  \| **Specify which outcome is being assessed for risk of bias** \| CKD \| \| --- \| --- \|  \| **Specify the numerical result being assessed.** In case of multiple alternative analyses being presented, specify the numeric result (e.g. RR = 1.52 (95% CI 0.83 to 2.77) and/or a reference (e.g. to a table, figure or paragraph) that uniquely defines the result being assessed. \| eGFR <50 or 60ml/min \| \| --- \| --- \|   **Is the review team’s aim for this result…?**   \| 🗌 \| to assess the effect of *assignment to intervention* (the ‘intention-to-treat’ effect) \| \| --- \| --- \| \| 🞫 \| to assess the effect of *adhering to intervention* (the ‘per-protocol’ effect) \|   **If the aim is to assess the effect of *adhering to intervention***, select the deviations from intended intervention that should be addressed (at least one must be checked):  □ occurrence of non-protocol interventions  🞫 failures in implementing the intervention that could have affected the outcome  🞫 non-adherence to their assigned intervention by trial participants  **Which of the following sources were obtained to help inform the risk-of-bias assessment? (tick as many as apply)**  🞫 Journal article(s) with results of the trial  □ Trial protocol  □ Statistical analysis plan (SAP)  □ Non-commercial trial registry record (e.g. ClinicalTrials.gov record)  □ Company-owned trial registry record (e.g. GSK Clinical Study Register record)  □ “Grey literature” (e.g. unpublished thesis)  □ Conference abstract(s) about the trial  □ Regulatory document (e.g. Clinical Study Report, Drug Approval Package)  □ Research ethics application  □ Grant database summary (e.g. NIH RePORTER or Research Councils UK Gateway to Research)  □ Personal communication with trialist  □ Personal communication with the sponsor |
| --- | --- | --- | --- | --- | --- | --- | --- | --- | --- | --- | --- | --- | --- | --- | --- | --- | --- | --- | --- | --- |

Risk of bias assessment

Responses underlined in green are potential markers for low risk of bias, and responses in red are potential markers for a risk of bias. Where questions relate only to sign posts to other questions, no formatting is used.

**Domain 1: Risk of bias arising from the randomization process**

| **Signalling questions** | **Comments** | **Response options** |
| --- | --- | --- |
| **1.1 Was the allocation sequence random?** | NA  NA | NA |
| **1.2 Was the allocation sequence concealed until participants were enrolled and assigned to interventions?** |  | NA |
| **1.3 Did baseline differences between intervention groups suggest a problem with the randomization process?** | NA | NA |
| **Risk-of-bias judgement** |  | High risk |
| Optional: What is the predicted direction of bias arising from the randomization process? |  | Unpredictable |

Domain 2: Risk of bias due to deviations from the intended interventions (*effect of assignment to intervention*)

| **Signalling questions** | **Comments** | **Response options** |
| --- | --- | --- |
| **2.1. Were participants aware of their assigned intervention during the trial?** | Oen label trial | Y |
| **2.2. Were carers and people delivering the interventions aware of participants' assigned intervention during the trial?** |  | Y |
| **2.3. If Y/PY/NI to 2.1 or 2.2: Were there deviations from the intended intervention that arose because of the trial context?** | No | N |
| **2.4 If Y/PY to 2.3: Were these deviations likely to have affected the outcome?** |  | NA |
| **2.5. If Y/PY/NI to 2.4: Were these deviations from intended intervention balanced between groups?** |  | NA |
| **2.6 Was an appropriate analysis used to estimate the effect of assignment to intervention?** | No information | NI |
| **2.7 If N/PN/NI to 2.6: Was there potential for a substantial impact (on the result) of the failure to analyse participants in the group to which they were randomized?** |  | NA |
| **Risk-of-bias judgement** |  | Low |
| Optional: What is the predicted direction of bias due to deviations from intended interventions? |  | NA |

Domain 2: Risk of bias due to deviations from the intended interventions (*effect of adhering to intervention*)

| **Signalling questions** | **Comments** | **Response options** |
| --- | --- | --- |
| **2.1. Were participants aware of their assigned intervention during the trial?** | Yes  Yes | Y |
| **2.2. Were carers and people delivering the interventions aware of participants' assigned intervention during the trial?** |  | Y |
| **2.3. [If applicable:] If Y/PY/NI to 2.1 or 2.2: Were important non-protocol interventions balanced across intervention groups?** | NA | NA |
| **2.4. [If applicable:] Were there failures in implementing the intervention that could have affected the outcome?** | No | PN/N |
| **2.5. [If applicable:] Was there non-adherence to the assigned intervention regimen that could have affected participants’ outcomes?** | No information | PN |
| **2.6. If N/PN/NI to 2.3, or Y/PY/NI to 2.4 or 2.5: Was an appropriate analysis used to estimate the effect of adhering to the intervention?** | No information | NI |
| **Risk-of-bias judgement** |  | Low |
| Optional: What is the predicted direction of bias due to deviations from intended interventions? |  | NA |

Domain 3: Missing outcome data

| **Signalling questions** | **Comments** | **Response options** |
| --- | --- | --- |
| **3.1 Were data for this outcome available for all, or nearly all, participants randomized?** | >5% lost follow up | PN |
| **3.2 If N/PN/NI to 3.1: Is there evidence that the result was not biased by missing outcome data?** | No information | PN |
| **3.3 If N/PN to 3.2: Could missingness in the outcome depend on its true value?** | No  NA | N |
| **3.4 If Y/PY/NI to 3.3: Is it likely that missingness in the outcome depended on its true value?** |  | NA |
| **Risk-of-bias judgement** |  | Low |
| Optional: What is the predicted direction of bias due to missing outcome data? |  | NA |

Domain 4: Risk of bias in measurement of the outcome

| **Signalling questions** | **Comments** | **Response options** |
| --- | --- | --- |
| **4.1 Was the method of measuring the outcome inappropriate?** | CKD diagnosed by eGFR <60ml/min estimated by MDRD | N |
| **4.2 Could measurement or ascertainment of the outcome have differed between intervention groups?** | No | N |
| **4.3 If N/PN/NI to 4.1 and 4.2: Were outcome assessors aware of the intervention received by study participants?** | Probably yes as it is open label trial | Y |
| **4.4 If Y/PY/NI to 4.3: Could assessment of the outcome have been influenced by knowledge of intervention received?** | No information  NA | PN |
| **4.5 If Y/PY/NI to 4.4: Is it likely that assessment of the outcome was influenced by knowledge of intervention received?** |  | NA |
| **Risk-of-bias judgement** |  | Low |
| Optional: What is the predicted direction of bias in measurement of the outcome? |  | NA |

Domain 5: Risk of bias in selection of the reported result

| **Signalling questions** | **Comments** | **Response options** |
| --- | --- | --- |
| **5.1 Were the data that produced this result analysed in accordance with a pre-specified analysis plan that was finalized before unblinded outcome data were available for analysis?** | Yes | Y |
| **Is the numerical result being assessed likely to have been selected, on the basis of the results, from...** |  |  |
| **5.2. ... multiple eligible outcome measurements (e.g. scales, definitions, time points) within the outcome domain?** | No | N |
| **5.3 ... multiple eligible analyses of the data?** | No | N |
| **Risk-of-bias judgement** |  | Low |
| Optional: What is the predicted direction of bias due to selection of the reported result? |  | NA |

Overall risk of bias

| **Risk-of-bias judgement** | Domain 1 has high risk of bias; other domains have low risk | High risk |
| --- | --- | --- |
| Optional: What is the overall predicted direction of bias for this outcome? |  | NA |


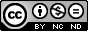


This work is licensed under a [Creative Commons Attribution-NonCommercial-NoDerivatives 4.0 International License](http://creativecommons.org/licenses/by-nc-nd/4.0/)
