# Supplementary material for: Chronic kidney disease among people living with HIV on TDF based regimen: A systematic review and meta-analysis
Source: PLoS One. 2025 Feb 6;20(2):e0318068. doi: 10.1371/journal.pone.0318068 (PMC11801554; doi:10.1371/journal.pone.0318068)
Supplement: S4 Table — (DOCX) [file pone.0318068.s004.docx]

**S4:** The risk of bias assessment tool results for the included studies.

| **Author** | **Representation** | **Sampling** | **Random selection** | **Non-response bias** | **Data collected** | **Case definition** | **Reliability &validity of tool** | **Mode of data collection** | **Length prevalence period** | **Numerator & denominator** | **The overall risk of bias** |
| --- | --- | --- | --- | --- | --- | --- | --- | --- | --- | --- | --- |
| Cournil A et al. 2017 | **Yes** | Yes | Yes | Yes | Yes | Yes | Yes | Yes | Yes | Yes | Low risk |
| Mwafongo A et al., 2015 | **No** | Yes | Yes | Yes | Yes | Yes | Yes | Yes | Yes | Yes | Low risk |
| Kalemeera F et al., 2020 | Yes | Yes | Yes | Yes | Yes | Yes | Yes | Yes | Yes | Yes | Low risk |
| Nyende L et al., 2020 | Yes | Yes | No | Yes | Yes | Yes | Yes | Yes | Yes | Yes | Low risk |
| Nartey ET et al., 2019 | Yes | Yes | Yes | Yes | Yes | Yes | Yes | Yes | Yes | Yes | Low risk |
| Ojen BV et al., 2018 | Yes | No | Yes | Yes | Yes | Yes | Yes | Yes | Yes | Yes | Low risk |
| Zachor H et al., 2016 | Yes | No | Yes | Yes | Yes | Yes | Yes | Yes | Yes | Yes | Low risk |
| Chikwapulo B et al., 2018 | Yes | No | Yes | Yes | Yes | Yes | Yes | Yes | Yes | Yes | Low risk |
| Bock P et al., 2019 | Yes | No | Yes | Yes | Yes | Yes | Yes | Yes | No | Yes | Low risk |
| Neary M et al., 2020 | Yes | No | Yes | Yes | Yes | Yes | Yes | Yes | Yes | Yes | Low risk |
| Belete AM et al., 2021 | Yes | No | Yes | Yes | Yes | Yes | Yes | Yes | No | Yes | Low risk |
| Debeb SG et al., 2021 | Yes | Yes | Yes | Yes | Yes | Yes | Yes | Yes | Yes | Yes | Low risk |
| Fritzsche C et al.,2017 | Yes | No | Yes | No | Yes | Yes | Yes | Yes | No | Yes | moderate risk |
| Chadwick DR et al., 2015 | Yes | No | Yes | Yes | Yes | Yes | Yes | Yes | No | Yes | Low risk |
| Okpa HO et al., 2019 | No | No | Yes | Yes | Yes | Yes | Yes | Yes | No | Yes | Moderate risk |
| Pujari SN., 2014 | Yes | No | Yes | Yes | Yes | Yes | Yes | Yes | No | Yes | Low risk |
| Jotwani V et al., 2016 | No | No | Yes | Yes | Yes | Yes | Yes | Yes | No | Yes | Moderate risk |
| Visuthrankul J et al., 2021 | Yes | Yes | Yes | Yes | Yes | Yes | Yes | Yes | Yes | Yes | Low risk |
| Nishijima T et al., 2014 | Yes | No | Yes | Yes | Yes | Yes | Yes | Yes | Yes | Yes | Low risk |
| Kyaw NTT et al., 2015 | Yes | No | Yes | Yes | Yes | Yes | Yes | Yes | No | Yes | Low risk |
| O'Donnel EP et al., 2011 | Yes | No | Yes | Yes | Yes | Yes | Yes | Yes | Yes | Yes | Low risk |
| Nishijima T et al., 2011 | Yes | No | Yes | Yes | Yes | Yes | Yes | Yes | No | Yes | Low risk |
| Woolnough EL et al., 2018 | Yes | No | Yes | Yes | Yes | Yes | Yes | Yes | Yes | Yes | Low risk |
| Nishijima T et al., 2017 | Yes | No | Yes | Yes | Yes | Yes | Yes | Yes | Yes | No | Low risk |
| Obiri-Yeboah D et al., 2018 | Yes | Yes | Yes | Yes | Yes | Yes | Yes | Yes | No | Yes | Low risk |
| Gallant JE et al., 2009 | Yes | No | Yes | Yes | Yes | Yes | Yes | Yes | Yes | Yes | Low risk |
| Hsu R et al., 2020 | Yes | No | Yes | Yes | Yes | Yes | Yes | Yes | Yes | Yes | Low risk |
| Lapadula G et al., 2016 | Yes | No | Yes | Yes | Yes | Yes | Yes | Yes | Yes | Yes | Low risk |
| Morlat P et al., 2013 | Yes | No | Yes | Yes | Yes | Yes | Yes | Yes | Yes | Yes | Low risk |
| Nishijima T et al., 2016 | Yes | No | Yes | Yes | Yes | Yes | Yes | Yes | Yes | Yes | Low risk |
| Kim JH et al., 2022 | No | No | Yes | Yes | Yes | Yes | Yes | Yes | Yes | Yes | Low risk |
| Young et al., 2007 | Yes | No | Yes | Yes | Yes | Yes | Yes | Yes | Yes | Yes | Low risk |
| Feng L et al., 2022 | Yes | No | Yes | Yes | Yes | Yes | Yes | Yes | Yes | Yes | Low risk |
| Sutton SS et al., 2020 | Yes | No | Yes | Yes | Yes | Yes | Yes | Yes | Yes | Yes | Low risk |
| Cheung J et al., 2018 | Yes | No | Yes | Yes | Yes | Yes | Yes | Yes | Yes | Yes | Low risk |
| Tan LKK et al., 2009 | Yes | No | Yes | Yes | Yes | Yes | Yes | Yes | Yes | Yes | Low risk |
| Milazzo L et al., 2016 | Yes | No | Yes | Yes | Yes | Yes | Yes | Yes | Yes | Yes | Low risk |
| Calza L et al., 2014 | Yes | No | Yes | Yes | Yes | Yes | Yes | Yes | Yes | Yes | Low risk |
| Quesada PR et al., 2015 | Yes | No | Yes | Yes | Yes | Yes | Yes | Yes | Yes | Yes | Low risk |
| Low JZ et al., 2018 | Yes | No | Yes | Yes | Yes | Yes | Yes | Yes | Yes | Yes | Low risk |
| Campbell LJ et al.,2009 | Yes | No | Yes | Yes | Yes | Yes | Yes | Yes | Yes | Yes | Low risk |
| Chabala FW et al., 2021 | Yes | Yes | Yes | Yes | Yes | Yes | Yes | Yes | Yes | Yes | Low risk |
| Flandre P et al.,2016 | Yes | No | Yes | No | Yes | Yes | Yes | Yes | Yes | Yes | Low risk |
| Suzuki S et al.,2017 | Yes | No | Yes | Yes | Yes | Yes | Yes | Yes | Yes | Yes | Low risk |
| Domingo P et al.,2019 | Yes | No | Yes | Yes | Yes | Yes | Yes | Yes | Yes | Yes | Low risk |
| Chan A et al., 2019 | Yes | No | Yes | No | Yes | Yes | Yes | Yes | Yes | Yes | Low risk |
| Lee KH et al.,2017 | Yes | No | Yes | No | Yes | Yes | Yes | Yes | Yes | Yes | Low risk |
| Likanonsakul S et al.,2016 | Yes | No | Yes | Yes | Yes | Yes | Yes | Yes | Yes | Yes | Low risk |
| Paengsai N et al.,2022 | Yes | No | Yes | Yes | Yes | Yes | Yes | Yes | Yes | Yes | Low risk |
| Ando M et al., 2011 | Yes | No | Yes | Yes | Yes | Yes | Yes | Yes | Yes | Yes | Low risk |
| Chua AC et al., 2012 | Yes | No | Yes | Yes | Yes | Yes | Yes | Yes | Yes | Yes | Low risk |
| Nishijima T et al., 2015 | Yes | No | Yes | Yes | Yes | Yes | Yes | Yes | Yes | Yes | Low risk |
| Ahmed E et al., 2020 | Yes | Yes | Yes | Yes | Yes | Yes | Yes | Yes | No | Yes | Low risk |
| Yazie TS et al., 2019 | Yes | No | Yes | Yes | Yes | Yes | Yes | Yes | Yes | Yes | Low risk |
| Huang Y et al., 2017 | Yes | No | Yes | Yes | Yes | Yes | Yes | Yes | Yes | Yes | Low risk |
| Calza L et al.,2013 | Yes | No | Yes | Yes | Yes | Yes | Yes | Yes | Yes | Yes | Low risk |
| Yang J et al., 2019 | Yes | No | Yes | Yes | Yes | Yes | Yes | Yes | Yes | Yes | Low risk |
| Juega-Mariño J et al., 2017 | Yes | No | Yes | Yes | Yes | Yes | Yes | Yes | No | Yes | Low risk |
| Mwemezi O et al., 2020 | Yes | Yes | Yes | Yes | Yes | Yes | Yes | Yes | No | Yes | Low risk |
| Reynes J et al., 2013 | Yes | No | Yes | Yes | Yes | Yes | Yes | Yes | No | Yes | Low risk |
| Monteagudo-Chu et al., 2012 | Yes | No | Yes | Yes | Yes | Yes | Yes | Yes | Yes | Yes | Low risk |
| Medland NA et al., 2017 | Yes | No | Yes | Yes | Yes | Yes | Yes | Yes | Yes | Yes | Low risk |
| Suppadungsuk S et al., 2022 | Yes | No | Yes | Yes | Yes | Yes | Yes | Yes | Yes | Yes | Low risk |
| Pedrol E et al., 2015 | Yes | No | Yes | Yes | Yes | Yes | Yes | Yes | Yes | Yes | Low risk |
| Kalemeera F et al., 2023 | Yes | No | Yes | Yes | Yes | Yes | Yes | Yes | Yes | Yes | Low risk |
| Joshi et al., 2019 | Yes | No | Yes | Yes | Yes | Yes | Yes | Yes | Yes | Yes | Low risk |
| Hoang C et al., 2020 | Yes | Yes | Yes | Yes | Yes | Yes | Yes | Yes | Yes | Yes | Low risk |
| Mocroft A et al., 2015 | Yes | Yes | Yes | Yes | Yes | Yes | Yes | Yes | Yes | Yes | Low risk |
| Crum-Cianflone N et al., 2010 | Yes | No | Yes | Yes | Yes | Yes | Yes | Yes | Yes | Yes | Low risk |
| Liu F et al., 2021 | Yes | No | Yes | Yes | Yes | Yes | Yes | Yes | Yes | Yes | Low risk |

- **Note**

**Hoy D et al., 2012**. Risk of bias assessment tool: Yes (low risk); No (high risk)

1. Representation: Was the study population a close representation of the national population?

2. Sampling: Was the sampling frame a true or close representation of the target population?

3. Random selection: Was some form of random selection used to select the sample OR was a census undertaken?

4. Non-response bias: Was the likelihood of non-response bias minimal?

5. Data collection: Were data collected directly from the subjects?

6. Case definition: Was an acceptable case definition used in the study?

7. Reliability and validity of study tool: Was the study instrument that measured the parameter of interest show to have reliability and validity?

8. Data collection: Was the same mode of data collection used for all subjects?

9. Prevalence period: Was the length of the prevalence period for the parameter of interest appropriate?

10. Numerators and denominators: Were the numerator(s) and denominator(s) for the parameter of interest appropriate?

The overall risk of bias scored based on the number of high risk of bias per study: low risk (≥8), moderate risk (5–7), and high risk (≤4)
