# Supplementary material for: Chronic kidney disease among people living with HIV on TDF based regimen: A systematic review and meta-analysis
Source: PLoS One. 2025 Feb 6;20(2):e0318068. doi: 10.1371/journal.pone.0318068 (PMC11801554; doi:10.1371/journal.pone.0318068)
Supplement: Attachment — Submitted filename: Response to reviewers.docx [file pone.0318068.s007.docx]

To The Editor, Reviewers

PLOS ONE

Date: Nov 26, 2024

**Subject: Addressing Editor/Reviewer Comments for PONE-D-24-20050**

**Dear Editor, Reviewers,**

We would like forwarding our great thanks for your crucial feedback to improve the status of the paper. We took long time to address the comments provided. Below, we are providing responses and actions to address each comment in a step-by-step way and in detail of our best ability. The responses are highlighted in blue.

Kind regards,

Authors of PONE-D-24-20050

PONE-D-24-20050

Chronic kidney disease among people living with HIV on TDF based regimen: A Systematic review and meta-analysis

PLOS ONE

Dear Dr. Yazie

Thank you for submitting your manuscript to PLOS ONE. After careful consideration, we feel that it has merit but does not fully meet PLOS ONE’s publication criteria as it currently stands. Therefore, we invite you to submit a revised version of the manuscript that addresses the points raised during the review process.

==============================
**ACADEMIC EDITOR:**

Please review the syntax and the language of the manuscript

Please submit your revised manuscript by Nov 28 2024 11:59PM. If you will need more time than this to complete your revisions, please reply to this message or contact the journal office at [plosone@plos.org](mailto:plosone@plos.org). When you're ready to submit your revision, log on to <https://www.editorialmanager.com/pone/> and select the 'Submissions Needing Revision' folder to locate your manuscript file.

Please include the following items when submitting your revised manuscript:

- A rebuttal letter that responds to each point raised by the academic editor and reviewer(s). You should upload this letter as a separate file labeled 'Response to Reviewers'.
  - Included
- A marked-up copy of your manuscript that highlights changes made to the original version. You should upload this as a separate file labeled 'Revised Manuscript with Track Changes'.
  - Included
- An unmarked version of your revised paper without tracked changes. You should upload this as a separate file labeled 'Manuscript'.
  - Included

If you would like to make changes to your financial disclosure, please include your updated statement in your cover letter. Guidelines for resubmitting your figure files are available below the reviewer comments at the end of this letter.

If applicable, we recommend that you deposit your laboratory protocols in [protocols.io](http://protocols.io/) to enhance the reproducibility of your results. Protocols.io assigns your protocol its own identifier (DOI) so that it can be cited independently in the future. For instructions see: [https://journals.plos.org/plosone/s/submission-guidelines#loc-laboratory-protocols](https://c05y1x9s.r.us-east-2.awstrack.me/L0/https:%2F%2Fjournals.plos.org%2Fplosone%2Fs%2Fsubmission-guidelines%23loc-laboratory-protocols/1/010f019289d50da4-2360828d-0e06-4652-8cf2-e5aab8fe5a06-000000/PImxcc_SbbJPvm2ViGid2FxmuGc=180). Additionally, PLOS ONE offers an option for publishing peer-reviewed Lab Protocol articles, which describe protocols hosted on [protocols.io](http://protocols.io/). Read more information on sharing protocols at [https://plos.org/protocols?utm_medium=editorial-email&utm_source=authorletters&utm_campaign=protocols](https://c05y1x9s.r.us-east-2.awstrack.me/L0/https:%2F%2Fplos.org%2Fprotocols%3Futm_medium=editorial-email%26utm_source=authorletters%26utm_campaign=protocols/1/010f019289d50da4-2360828d-0e06-4652-8cf2-e5aab8fe5a06-000000/kQusqVAL1JrUhP9SEzHuU-owSdk=180).

We look forward to receiving your revised manuscript.

Kind regards,

Tarek Samy Abdelaziz, MD
Academic Editor
PLOS ONE

Journal Requirements:

When submitting your revision, we need you to address these additional requirements.

1. Please ensure that your manuscript meets PLOS ONE's style requirements, including those for file naming. The PLOS ONE style templates can be found at

<https://journals.plos.org/plosone/s/file?id=wjVg/PLOSOne_formatting_sample_main_body.pdf> and

[https://journals.plos.org/plosone/s/file?id=ba62/PLOSOne_formatting_sample_title_authors_affiliations.pdf](https://c05y1x9s.r.us-east-2.awstrack.me/L0/https:%2F%2Fjournals.plos.org%2Fplosone%2Fs%2Ffile%3Fid=ba62%2FPLOSOne_formatting_sample_title_authors_affiliations.pdf/1/010f019289d50da4-2360828d-0e06-4652-8cf2-e5aab8fe5a06-000000/pzUOcAQP6-MsEowbxyKHlZwrF_c=180).

- We have checked that the manuscript meets the PLOS ONE journal requirements.

2. Please include captions for your Supporting Information files at the end of your manuscript, and update any in-text citations to match accordingly. Please see our Supporting Information guidelines for more information: [http://journals.plos.org/plosone/s/supporting-information](http://c05y1x9s.r.us-east-2.awstrack.me/L0/http:%2F%2Fjournals.plos.org%2Fplosone%2Fs%2Fsupporting-information/1/010f019289d50da4-2360828d-0e06-4652-8cf2-e5aab8fe5a06-000000/03ktoFeC51Lt_TDssjf2_kGyMmA=180).

- We have included captions for all supporting information.

3. As required by our policy on Data Availability, please ensure your manuscript or supplementary information includes the following:

A numbered table of all studies identified in the literature search, including those that were excluded from the analyses.

- We have included a numbered table in excel of all studies identified in literature search (S5 Table). Reasons of exclusion have been included in this table of excel.

For every excluded study, the table should list the reason(s) for exclusion.

- We have included reasons of exclusion for all excluded studies that are identified in literature search (S5 Table).

If any of the included studies are unpublished, include a link (URL) to the primary source or detailed information about how the content can be accessed.

- This is not applicable in this review, as no unpublished studies were identified during the literature search.

A table of all data extracted from the primary research sources for the systematic review and/or meta-analysis. The table must include the following information for each study:

Name of data extractors and date of data extraction

Confirmation that the study was eligible to be included in the review.

All data extracted from each study for the reported systematic review and/or meta-analysis that would be needed to replicate your analyses.

- We have included it as per suggestion (S6 Table).

If data or supporting information were obtained from another source (e.g. correspondence with the author of the original research article), please provide the source of data and dates on which the data/information were obtained by your research group.

- We attempted to contact the corresponding authors obtain missing data for some studies, but authors did not respond to our requests. Therefore, in this review, there are no data obtained from another sources.

If applicable for your analysis, a table showing the completed risk of bias and quality/certainty assessments for each study or outcome.  Please ensure this is provided for each domain or parameter assessed. For example, if you used the Cochrane risk-of-bias tool for randomized trials, provide answers to each of the signalling questions for each study. If you used GRADE to assess certainty of evidence, provide judgements about each of the quality of evidence factor. This should be provided for each outcome.

- We have included the methodological quality assessment results for randomized clinical trials based on Cochrane risk-of-bias tool for randomized trials. Each domain has been assessed and also answers were provided for each signalling question within each domain (included under S3 File).

An explanation of how missing data were handled.

This information can be included in the main text, supplementary information, or relevant data repository. Please note that providing these underlying data is a requirement for publication in this journal, and if these data are not provided your manuscript might be rejected.

- In this review, we used a complete-case analysis approach to include only studies that had complete data for the outcome of interest. For studies with missing data, we tried to contact the corresponding authors to obtain the missing information. However, despite these efforts, none of them responded to our requests.

4. Please review your reference list to ensure that it is complete and correct. If you have cited papers that have been retracted, please include the rationale for doing so in the manuscript text, or remove these references and replace them with relevant current references. Any changes to the reference list should be mentioned in the rebuttal letter that accompanies your revised manuscript. If you need to cite a retracted article, indicate the article’s retracted status in the References list and also include a citation and full reference for the retraction notice.

- We have checked all references for their completeness and correctness. No retracted articles found in the included references.


  [Note: HTML markup is below. Please do not edit.]

  Reviewers' comments:

  Reviewer's Responses to Questions

**Comments to the Author**

1. Is the manuscript technically sound, and do the data support the conclusions?

The manuscript must describe a technically sound piece of scientific research with data that supports the conclusions. Experiments must have been conducted rigorously, with appropriate controls, replication, and sample sizes. The conclusions must be drawn appropriately based on the data presented.

Reviewer #1: Yes

2. Has the statistical analysis been performed appropriately and rigorously?

Reviewer #1: Yes

3. Have the authors made all data underlying the findings in their manuscript fully available?

The [PLOS Data policy](http://c05y1x9s.r.us-east-2.awstrack.me/L0/http:%2F%2Fwww.plosone.org%2Fstatic%2Fpolicies.action%23sharing/1/010f019289d50da4-2360828d-0e06-4652-8cf2-e5aab8fe5a06-000000/241vRgXmtAZu6-HrR9WzELvTGo8=180) requires authors to make all data underlying the findings described in their manuscript fully available without restriction, with rare exception (please refer to the Data Availability Statement in the manuscript PDF file). The data should be provided as part of the manuscript or its supporting information, or deposited to a public repository. For example, in addition to summary statistics, the data points behind means, medians and variance measures should be available. If there are restrictions on publicly sharing data—e.g. participant privacy or use of data from a third party—those must be specified.

Reviewer #1: Yes

4. Is the manuscript presented in an intelligible fashion and written in standard English?

PLOS ONE does not copyedit accepted manuscripts, so the language in submitted articles must be clear, correct, and unambiguous. Any typographical or grammatical errors should be corrected at revision, so please note any specific errors here.

Reviewer #1: Yes

5. Review Comments to the Author

Please use the space provided to explain your answers to the questions above. You may also include additional comments for the author, including concerns about dual publication, research ethics, or publication ethics. (Please upload your review as an attachment if it exceeds 20,000 characters)

Reviewer #1: The manuscript by Yazie et al. “Chronic kidney disease among people living with HIV on TDF based regimen: A Systematic review and meta-analysis” looks interesting. This systematic review article aims to determine the prevalence of CKD among PLWHIV receiving a TDF based regimen using publications between January 2000 and May 2024. The meta-analysis in this article included 69 studies with 88299 participants and found that the pooled prevalence of CKD was 7% (95% CI:6-8) whereas CD4 count was less than 200 copies per milliliter in females associated with CKD. The authors concluded that the magnitude of CKD across the globe is high in people with HIV and who have received TDF based regimen.
The manuscript is well written and provides enough strategies to fulfill its aims, though language may need some polishing for better flow. In conclusion, I support publication of this systematic review and believe that it would be interesting for researchers working in the field.

- Dear Reviewer, we thank you for your encouragement and helpful comments. We have done our best to improve the language of this paper.

6. PLOS authors have the option to publish the peer review history of their article ([what does this mean?](https://c05y1x9s.r.us-east-2.awstrack.me/L0/https:%2F%2Fjournals.plos.org%2Fplosone%2Fs%2Feditorial-and-peer-review-process%23loc-peer-review-history/1/010f019289d50da4-2360828d-0e06-4652-8cf2-e5aab8fe5a06-000000/4xrLX_mjX0TKA1d-N3-y21EFOio=180)). If published, this will include your full peer review and any attached files.


If you choose “no”, your identity will remain anonymous but your review may still be made public.


**Do you want your identity to be public for this peer review?** For information about this choice, including consent withdrawal, please see our [Privacy Policy](https://c05y1x9s.r.us-east-2.awstrack.me/L0/https:%2F%2Fwww.plos.org%2Fprivacy-policy/1/010f019289d50da4-2360828d-0e06-4652-8cf2-e5aab8fe5a06-000000/7uTQtco7SKFRDOQ1pJC9xr8D8ks=180).

Reviewer #1: No

[NOTE: If reviewer comments were submitted as an attachment file, they will be attached to this email and accessible via the submission site. Please log into your account, locate the manuscript record, and check for the action link "View Attachments". If this link does not appear, there are no attachment files.]

While revising your submission, please upload your figure files to the Preflight Analysis and Conversion Engine (PACE) digital diagnostic tool, [https://pacev2.apexcovantage.com/](https://c05y1x9s.r.us-east-2.awstrack.me/L0/https:%2F%2Fpacev2.apexcovantage.com%2F/1/010f019289d50da4-2360828d-0e06-4652-8cf2-e5aab8fe5a06-000000/4jfa9UoS7vohwDLbSsVYXWH1LOM=180). PACE helps ensure that figures meet PLOS requirements. To use PACE, you must first register as a user. Registration is free. Then, login and navigate to the UPLOAD tab, where you will find detailed instructions on how to use the tool. If you encounter any issues or have any questions when using PACE, please email PLOS at [figures@plos.org](mailto:figures@plos.org). Please note that Supporting Information files do not need this step.

*In compliance with data protection regulations, you may request that we remove your personal registration details at any time.* [*(Remove my information/details)*](https://c05y1x9s.r.us-east-2.awstrack.me/L0/https:%2F%2Fwww.editorialmanager.com%2Fpone%2Flogin.asp%3Fa=r/1/010f019289d50da4-2360828d-0e06-4652-8cf2-e5aab8fe5a06-000000/Qp2yCN3wk3NQVoz6EECEN6kmdGs=180)*. Please contact the publication office if you have any questions.*
